# Synthesis and Characterization of a Metalloid Ge_6_ Cluster with Bulky Amide Ligands

**DOI:** 10.3390/ma19122516

**Published:** 2026-06-11

**Authors:** Jingjing Liu, Xiaoting Liu, Bin Zhang, Caiting Ji, Xiaohui Sun, Wenyuan Wang, Xiaoxu Bo

**Affiliations:** 1 School of Energy Engineering, Shanxi College of Technology, Shuozhou 036000, China; liujingjing@sxct.edu.cn (J.L.); liuxiaoting@sxct.edu.cn (X.L.); zhangbin@sxct.edu.cn (B.Z.); jicaiting@sxct.edu.cn (C.J.); xiaohuisun_sxct@163.com (X.S.); 2 Key Laboratory of Synthetic and Natural Functional Molecule of Ministry of Education, College of Chemistry and Materials Science, Northwest University, Xi’an 710127, China; 3 Department of Agriculture and Biotechnology, Wenzhou Vocational College of Science and Technology, Wenzhou 325006, China

**Keywords:** metalloid germanium cluster, bulky amido group, butterfly-shaped geometry, bis(amido)-substituted germylene

## Abstract

This article details the synthesis and structural characterization of a new metalloid germanium cluster **3** with bulky amide ligands. The cluster features a Ge_6_ core stabilized by four -N(Si*^t^*BuMe_2_)_2_ ligands and was obtained via reduction of the amido trichlorogermane **2** using potassium chips in toluene. Single-crystal X-ray diffraction analysis revealed that the Ge_6_ core adopts a butterfly-shaped geometry with a Ge-Ge dumbbell unit, which contains two unsubstituted germanium atoms exhibiting prominent lone-pair characteristics. The Ge_6_ core can also be classified as a *nido* cluster, with a cluster-bonding-electron count of 16, perfectly satisfying the 2n + 4 electron-counting rule. Combining the structural features of this *nido* cluster with the bond length distribution in the folded four-membered ring suggests that the Ge4 ring features a certain degree of electron delocalization. Additionally, two bis(amido)-substituted germylenes (**4** and **6**) were isolated and structurally characterized. They exhibit analogous structural features, with each germanium center adopting a two-coordinate V-shaped configuration, the Ge–N bond lengths being very similar, and the nitrogen atoms adopting a planar triangular geometry. Notably, compound **6**, bearing bulkier -N(Si*^i^*Pr_3_)_2_ substituents, exhibits a significantly larger N-Ge-N bond angle (120.58°) compared to the corresponding value of 113.54° observed for compound **4** with -N(Si*^t^*BuMe_2_)_2_ substituents. This clearly demonstrates that the steric bulk of the substituents exerts a remarkable influence on the molecular geometry and *σ*-donor ability of the lone pairs on germanium centers.

## 1. Introduction

Metalloid clusters are defined as a class of compounds wherein the amount of metal–metal contact exceeds that of metal–ligand bonds; additionally, they are characterized by the presence of metal atoms that participate exclusively in metal–metal interactions [1]. These unsubstituted or so-called “naked” metal atoms often adopt inverted tetrahedral geometries [2,3,4]. This concept first emerged in the chemistry of Al and Ga [5,6] and was later extended to the heavier Group 14 elements, giving rise to a series of metalloid clusters of silicon [7,8,9,10], germanium [11,12,13,14,15], and tin [16,17,18,19,20] with nuclearities ranging from five- to eight-atom frameworks. The advent of these species elegantly confirms that the heavier Group 14 elements tend to form spherical frameworks bearing numerous *σ* bonds [21,22], and such clusters have found promising applications in materials science, biomedicine, and modern electronic information technologies [23,24,25]. Most of these clusters are stabilized by alkyl, aryl, or silyl substituents. Among them, the most well-known stable polyhedral clusters of the heavier Group 14 elements are ligand-free Zintl anions, such as M_9_^n−^ (M = Ge, Sn, or Pb—*n* = 2, 3, 4) [26]. Nevertheless, analogous cluster examples stabilized by amido ligands remain scarce.

**Scheme 1:**
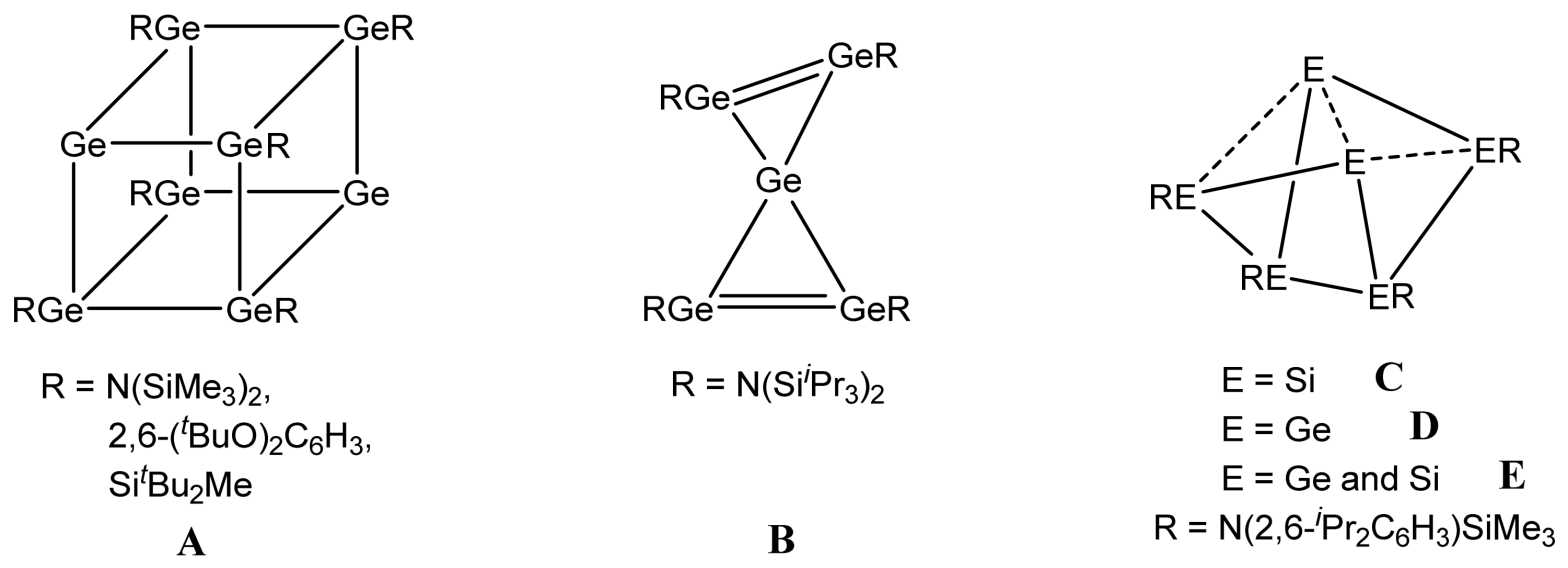
Amino-substituted ligand-free metalloid germanium clusters.

In 2006, Lappert and Power reported the first high-nuclearity unsaturated tin cluster, Sn_15_{N(SiMe_3_)Dipp}_6_ [19], stabilized by amino substituents. It was synthesized via reductive dehalogenation of the dimer [{N(SiMe_3_)Dipp}SnCl_2_]_2_, with KC_8_ serving as the reducing agent, in which the ligand-free Sn atoms formed a body-centered distorted cube. Although unsaturated amido-stabilized metalloid germanium clusters can be prepared via similar synthetic strategies, such synthetic routes are generally difficult to control [9,27,28,29] and prone to byproduct formation [30] and deliver low isolated yields, as exemplified by the Ge_8_ cubane **A** [31,32,33] and Ge_5_ spiropentadiene **B** [34]. Recently, there has been progress in this field. Lips and co-workers consecutively reported three structurally similar unsaturated amido-substituted hexanuclear metalloid clusters: silicon cluster **C** [8], germanium cluster **D** [13], and mixed silicon–germanium cluster **E** [35] (Scheme 1). In compound **D**, the Ge_6_ core bearing two unsubstituted Ge atoms is stabilized by four amido substituents. This Ge_6_ cluster adopts a butterfly-shaped structure capped by dumbbell-like moieties, and the two unsubstituted Ge atoms exhibit distinct lone-pair characteristics.

It is well known that extremely bulky monodentate amido protecting groups have been widely applied in the synthesis of low-coordinate complexes of main-group elements [36,37,38], transition metals [39,40,41], and f-block metals [42,43,44]. The steric hindrance of amido groups can significantly affect the polymerization behavior of such complexes. Herein, we report the synthesis and crystal structure of metalloid germanium cluster **3**, which is stabilized by four bulky amido substituents (-N(Si*^t^*BuMe_2_)_2_). To further understand the structural features of this new germanium cluster, we analyzed the molecular orbitals via density functional theory (DFT) computational calculations.

## 2. Materials and Methods

### 2.1. General Remarks

All experimental manipulations were performed under inert atmosphere of purified N_2_ or Ar using standard Schlenk lines and glovebox techniques. Glassware was heat-dried and cooled down under vacuum prior to use. All solvents were dried, distilled and deoxygenated. The solid reactants were weighed in the glove box, and liquid reagents were added with syringe or drip funnel under inert atmosphere. The starting materials GeCl_2_∙dioxane [45], KN(Si*^t^*BuMe_2_)_2_ [46] and KN(Si*^i^*Pr_3_)_2_ [47] were prepared according to procedures from the literature. Potassium was treated under vigorous stirring and refluxing in hexane under argon, and then cooled rapidly to form potassium grains. The ultra-fine potassium chips were screened and used for the reduction reactions. The ^1^H and ^13^C{^1^H} NMR spectra were recorded on JEOL ECZ 400R spectrometer (Tokyo, Japan). Elemental analysis (C, H, N) was performed on Perkin-Elmer 2400 CHN elemental analyzer (Waltham, MA, USA). Melting points were measured on TD-V20G melting point apparatus. The single-crystal X-ray diffractions were performed on Bruker D8 VENTURE PHOTON II detector (Karlsruhe, Germany) (compounds **2**, **4** and **6** at 150 K) with a Ga-target Liquid X-ray source (*λ* = 1.34138 Å). Compound **3** was measured on Bruker D8 Quest detector at 150 K with a Mo-Kα X-ray source (*λ* = 0.71073 Å).

### 2.2. Synthesis of Compound ***2***

A solution of KN(Si*^t^*BuMe_2_)_2_ (0.500 g, 1.76 mmol) in Et_2_O (20 mL) was added dropwise to a solution of GeCl_4_ (0.454 g, 2.12 mmol) in Et_2_O (15 mL) at −78 °C under vigorous stirring. The reaction mixture turned yellow, which was further stirred at this temperature for 5 h. All volatiles were removed under reduced pressure and the residue was extracted with hexane (20 mL). The extract was filtered, concentrated and stored at −20 °C in the freezer overnight, affording colorless crystals of **2** (0.590 g, 1.39 mmol, 79%). The crystal was measured by single-crystal X-ray diffraction analysis. **M.p.** 151–153 °C. **^1^H NMR** (400 MHz, C_6_D_6_, 298 K): *δ* = 0.33 (s, 12H, C(CH_3_)_3_*Me*_2_), 0.98 (s, 18H, C(*CH*_3_)_3_Me_2_). **^13^C NMR** (100 MHz, C_6_D_6_, 298 K): *δ* = 28.62 (C(CH_3_)_3_*Me*_2_), 41.93 (*C*(CH_3_)_3_Me_2_), 48.94 (C(*CH*_3_)_3_Me_2_). **^29^Si NMR** (600 MHz, C_6_D_6_, 298 K): *δ* = 10.12 (N-*Si^t^*BuMe_2_). **Anal. Calcd for** C_12_H_30_Cl_3_GeNSi_2_: C, 34.03; H, 7.14; N, 3.31. Found: C, 34.23; H, 7.16; N, 3.28.

### 2.3. Synthesis of Compounds ***3*** and ***4***

To a solution of **2** (0.370 g, 0.874 mmol) in toluene (10 mL) was added potassium chips (0.130 g, 3.320 mmol) at ambient temperature. After stirring for 72 h, the color of the reaction mixture changed from yellow-green to brownish purple. Volatiles were removed under reduced pressure and the residue was extracted with hexane (ca. 20 mL). The resulting suspension was filtered, and the concentrated clear filtrate was cooled to −20 °C for 24 h to afford reddish-brown strip crystals of **3** (0.097 g, 0.069 mmol, 47.4%). The supernatant was transferred, further concentrated and stored at −20 °C, and **4** was obtained as orange strip crystals (0.038 g, 0.068 mmol, 7.8%). Both were characterized by crystallographic analysis. **3**: **M.p.** 211–213 °C. **4**: **M.p.** 75–77 °C. **3**: **^1^H NMR** (400 MHz, C_6_D_6_, 298 K): *δ* = 0.54 (s, 48H, C(CH_3_)_3_*Me*_2_) (A), 0.55 (s, 48H, C(CH_3_)_3_*Me*_2_) (B), 1.18 (s, 72H, C(*CH*_3_)_3_Me_2_) (B), 1.19 (s, 72H, C(*CH_3_*)_3_Me_2_) (A). **4**: **^1^H NMR** (400 MHz, C_6_D_6_, 298 K): *δ* = 0.42 (s, 24H, C(CH_3_)_3_*Me*_2_), 1.07 (s, 36H, C(*CH*_3_)_3_Me_2_). **3**: **^13^C NMR** (100 MHz, C_6_D_6_, 298 K): *δ* = 20.60 (C(*C*H_3_)_3_Me_2_) (A), 20.86 (C(CH_3_)_3_*Me*_2_) (A), 21.60 (*C*(CH_3_)_3_Me_2_) (A), 29.25 (C(*C*H_3_)_3_Me_2_) (B), 29.93 (C(CH_3_)_3_*Me*_2_) (B), 30.31 (*C*(CH_3_)_3_Me_2_) (B). **4**: **^13^C NMR** (100 MHz, C_6_D_6_, 298 K): *δ* = 28.14 (C(CH_3_)_3_*Me*_2_), 42.09 (*C*(CH_3_)_3_Me_2_), 49.01 (C(*C*H_3_)_3_Me_2_). **3**: **^29^Si NMR** (600 MHz, C_6_D_6_, 298 K): *δ* = 6.15 (N-*Si^t^*BuMe_2_) (A), 6.63 (N-*Si^t^*BuMe_2_) (B). **4**: **^29^Si NMR** (600 MHz, C_6_D_6_, 298 K): *δ* = 5.17 (N-*Si^t^*BuMe_2_). **3**: **Anal. Calcd for** C_48_H_120_Ge_6_N_4_Si_8_: C, 40.77; H, 8.55; N, 3.96. Found: C, 41.02; H, 8.57; N, 3.90. **4**: **Anal. Calcd for** C_24_H_60_GeN_2_Si_4_: C, 51.32; H, 10.77; N, 4.99. Found: C, 51.42; H, 10.82; N, 4.96.

### 2.4. Synthesis of Compound ***6***

To the suspension of GeCl_2_∙dioxane (0.315 g, 1.36 mmol) in Et_2_O (15 mL) was added a solution of KN(Si*^i^*Pr_3_)_2_ (1.00 g, 2.72 mmol) in Et_2_O (15 mL) under vigorous stirring at room temperature. The reaction mixture was stirred for 12 h, resulting in a yellow slurry. The solvent was removed under reduced pressure, and the residue was extracted with hexane (ca. 20 mL). The concentrated filtrate was stored at −20 °C, yielding **6** as orange crystals (0.615 g, 0.842 mmol, 62%), which were suitable for the single-crystal X-ray diffraction analysis. **M.p.** 69–71 °C. **^1^H NMR** (400 MHz, C_6_D_6_, 298 K): *δ* = 1.36 (d, **^3^***J*_HH_ = 6.8 Hz, 72H, CH*Me*_2_), 1.50 (sept, **^3^***J*_HH_ = 6.8 Hz, 12H, C*H*Me_2_). **^13^C NMR** (100 MHz, C_6_D_6_, 298 K): *δ* = 18.59 (*C*HMe_2_), 21.10 (CH*Me*_2_). **^29^Si NMR** (600 MHz, C_6_D_6_, 298 K): *δ* = 6.84 (N-*Si^i^*Pr_3_). **Anal. Calcd for** C_36_H_84_GeN_2_Si_4_: C, 59.23; H, 11.60; N, 3.84. Found: C, 59.47; H, 11.68; N, 3.80.

## 3. Results and Discussion

### 3.1. NMR and Single-Crystal Structural Characterization of Trichlorogermane ***2***

**Scheme 2:**
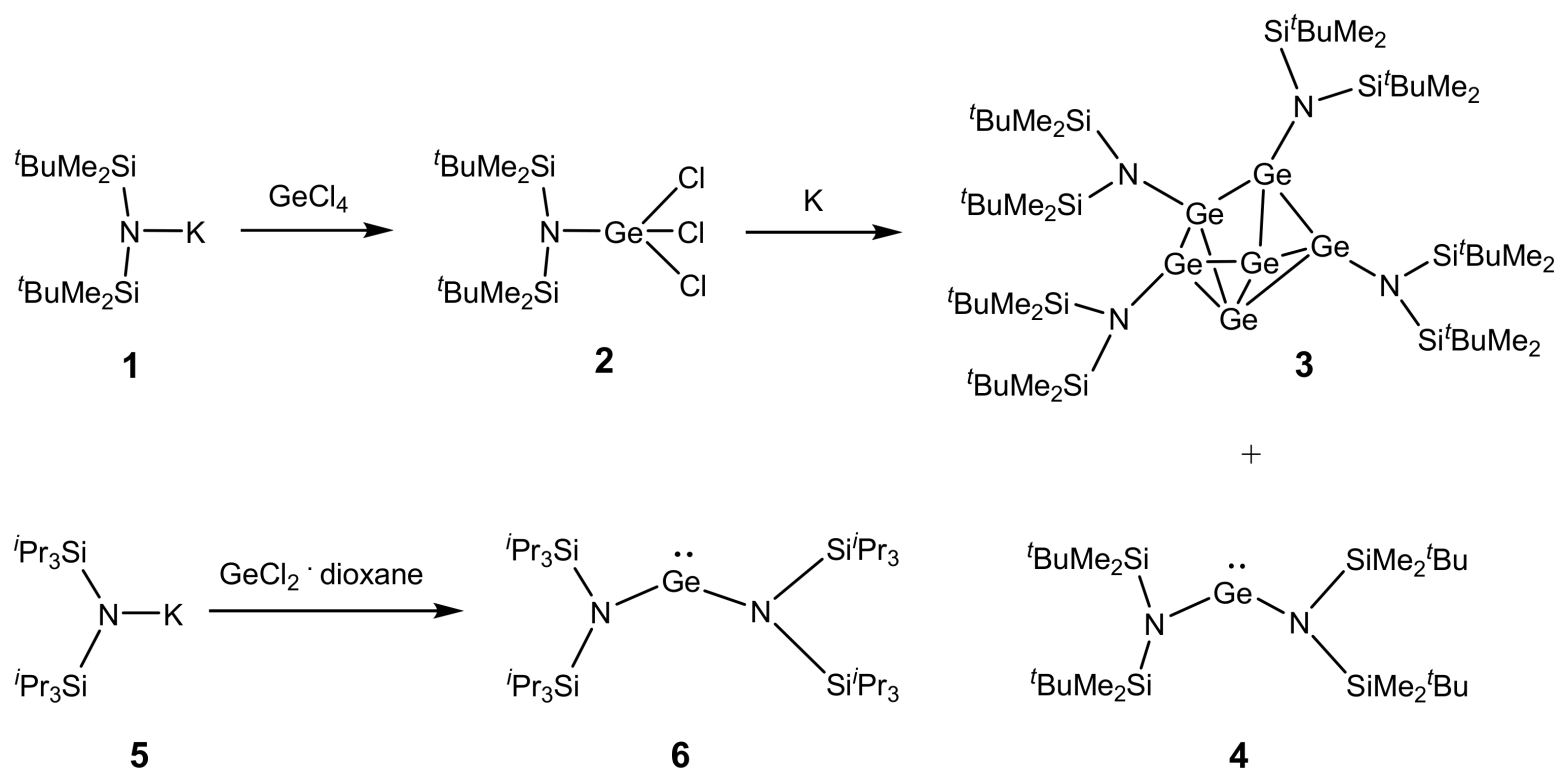
Synthesis of compounds **2**–**4** and **6**.

**Figure 1:**
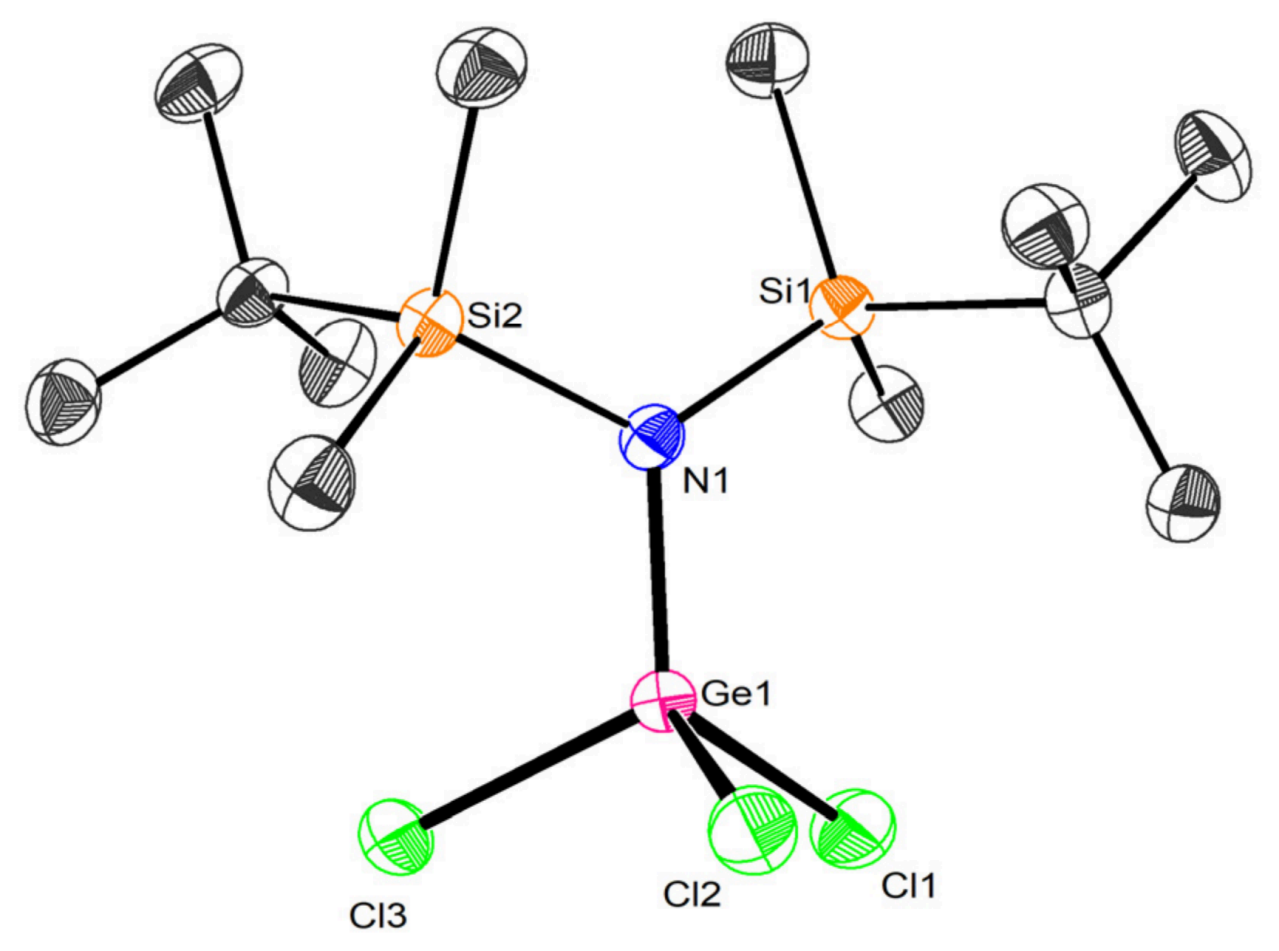
Molecular structure of compound **2**. Thermal ellipsoids are drawn at the 50% probability level. All hydrogen atoms are omitted for clarity. Selected bond lengths (Å) and angles (°): Ge1-Cl3 2.1357(9), Ge1-Cl2 2.1333(9), Ge1-Cl1 2.1272(10), Ge1-N1 1.7966(19), Si2-N1 1.799(2), Si1-N1 1.789(2), Cl2-Ge1-Cl3 105.71(4), Cl1-Ge1-Cl3 102.18(4), Cl1-Ge1-Cl2 103.94(4), N1-Ge1-Cl3 113.69(6), N1-Ge1-Cl2 112.78(6), N1-Ge1-Cl1 117.24(7), Si1-N1-Si2 123.48(11).

The reaction of bulky potassium amide **1** [46] with stoichiometric GeCl_4_ via salt metathesis afforded amido trichlorogermane **2** in a high yield (Scheme 2). The composition and constitution of **2** were fully characterized via ^1^H, ^13^C and ^29^Si NMR spectroscopy, elemental analysis, and single-crystal X-ray diffraction. The ^1^H NMR spectrum of **2** exhibits one singlet at *δ* = 0.33 ppm, assigned to methyl moieties, and a singlet at *δ* = 0.98 ppm for tert-butyl methyl protons. The molecular structure of **2** (Figure 1), determined via single-crystal X-ray diffraction, confirms that it is a monomeric four-coordinate amido trichlorogermane, in which Ge1 adopts a tetrahedral geometry with an average bond angle of 109.26°. The average Ge-Cl distance (2.1321(9) Å) and Ge1−N1 bond lengths (1.7966(19) Å) in **2** are shorter than the corresponding values in two-coordinate amido germylene chloride [34] and three-coordinate Ge^II^ species [48,49,50]. This contraction can presumably be attributed to stronger orbital interaction between the vacant 4p orbital of the Ge atom and the lone-pair electrons of the adjacent N and Cl atoms.

### 3.2. NMR and Single-Crystal Structural Characterization of Metalloid Germanium Cluster ***3***

As heavier Group 14 elements are less likely to form *π* bonds, we were curious to determine how the steric hindrance of bulky amido ligands influences the oligomerization of such complexes. The reduction of compound **2** with 3.8 equivalents of potassium chips in toluene led to the formation of the metalloid germanium cluster **3**, which precipitated as reddish-brown crystals from a hexane solution at −20 °C in a 47.4% yield. We propose that the reduction process is accompanied by the elimination of two equivalents of bis(amido)-substituted germylene, constituting a plausible formation pathway for cluster **3**. The supernatant was further concentrated and kept at −20 °C. Fortunately, we successfully isolated the bis(amido)-substituted germylene **4** as orange crystals in a 7.8% yield, and they were suitable for crystallographic analysis. Compound **4** was also successfully obtained by treating compound **1** with GeCl_2_·dioxane in a molar ratio of 2:1. Both compounds **3** and **4** were fully characterized via spectroscopy and single-crystal X-ray crystallography. The ^1^H NMR spectrum of compound **3** displays two distinct sets of signals. The signals at *δ* = 0.54 and 0.55 ppm were assigned to the methyl protons of the silyl groups, while those at *δ* = 1.18 and 1.19 ppm correspond to the methyl groups of the tert-butyl moieties. In the ^29^Si NMR spectrum of compound **3**, two sharp singlet resonances were observed at *δ* = 6.15 and 6.63 ppm, respectively. One set of signals arises from the amido substituents attached to the upper folded four-membered ring, and the other set originates from the amido substituents connected to the lower dumbbell unit. This indicates a dynamic exchange between the two chemically and magnetically different substituents. Such dynamic behavior is common in metalloid clusters [13]. The proposed process behind the molecular dynamics of compound **3** is shown in Figure S13.

**Figure 2:**
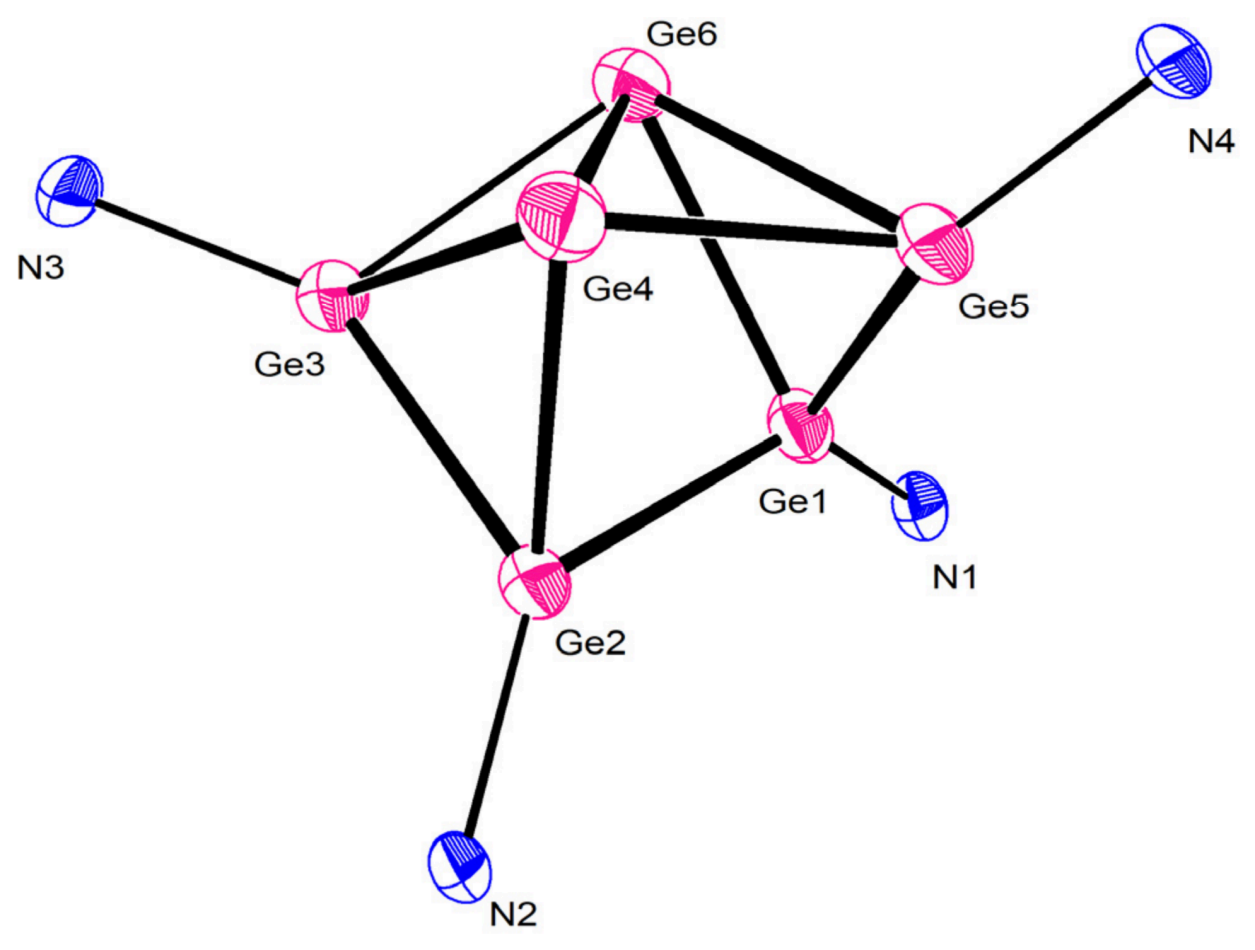
Molecular structure of compound **3**. Thermal ellipsoids are drawn at the 50% probability level. The Si*^t^*BuMe_2_ groups are omitted for clarity. Selected bond lengths (Å): Ge1-Ge2 2.6026(6), Ge1-Ge5 2.4824(5), Ge1-Ge6 2.6163(6), Ge2-Ge3 2.4914(5), Ge2-Ge4 2.6162(6), Ge3-Ge4 2.7439(6), Ge3-Ge6 2.4654(6), Ge5-Ge4 2.4676(6), Ge5-Ge6 2.7368(6), Ge4-Ge6 2.6820(6), Ge1-N1 1.897(3), Ge2-N2 1.901(3), Ge3-N3 1.892(2), Ge5-N4 1.897(3). Selected bond angles (°): Ge6-Ge4-Ge3 54.036(15), Ge5-Ge4-Ge6 64.050(16), Ge2-Ge4-Ge3 55.334(13), Ge5-Ge4-Ge2 81.987(17).

Furthermore, single-crystal X-ray diffraction analysis revealed that all germanium atoms in cluster **3** are four-coordinate (Figure 2). The Ge_6_ cluster core, which contains two unsubstituted germanium atoms, is surrounded by four -N(Si*^t^*BuMe_2_)_2_ substituents. Atoms Ge3, Ge4, Ge5, and Ge6 form a butterfly-shaped fragment that is connected to a dumbbell unit consisting of Ge1-Ge2. Within the folded four-membered unit, the bond lengths of Ge3-Ge6 and Ge4-Ge5 (2.4654(6) Å and 2.4676(6) Å, respectively) are close to the typical Ge-Ge single bond (2.445 Å) in elemental germanium and the corresponding bond lengths (2.4481(7) Å) in the metalloid germanium cluster **D**. In contrast, the Ge3-Ge4 and Ge5-Ge6 bond lengths are 2.7439(6) Å and 2.7368(6) Å, respectively, which are greater than the Ge-Ge single bonds in some Ge_2_Si_2_ species [51] and the germanium cation Ge_4_^2+^ [52], but slightly shorter than the corresponding bond length (2.7852(8) Å) in cluster **D**. The transannular distance between Ge4 and Ge6 is 2.6820(6) Å, which is shorter than the distances observed in cluster **D** (2.7268(11) Å) and other metalloid germanium clusters (2.829(5)–2.886(2) Å). The Ge2-Ge3 (2.4914(5) Å) and Ge1-Ge5 (2.4824(5) Å) bonds, which connect the folded four-membered unit to the Ge1-Ge2 dumbbell, both fall within the range of typical Ge-Ge single bonds. However, the Ge2-Ge4 (2.6162(6) Å) and Ge1-Ge6 (2.6163(6) Å) bonds are elongated relative to Ge-Ge single bonds, a phenomenon that is frequently observed in metalloid germanium clusters. The Ge-N bond lengths (1.892(2)–1.901(3) Å) are only somewhat longer than the corresponding bonds in precursor **2** and metalloid germanium cluster **D**, and all fall within the normal range for Ge-N single bonds [32].

More importantly, in metalloid germanium cluster **3**, the bond angles around the ligand-free Ge4 atoms are 55°, 82°, 54°, and 64°. Accordingly, the unsubstituted Ge atoms can be regarded as occupying the apical positions of a distorted square-based pyramid. This is a common feature of “naked” Ge atoms, as analyzed by Scheschkewitz [10]. The core of compound **3** can also be described as a twisted trigonal prism or a distorted octahedron with two edges missing.

From a cluster chemistry perspective, the Ge_6_ core can be formally classified as a *nido* cluster [53]. The cluster-bonding-electron count is 16, which perfectly satisfies the 2n + 4 electron-counting rule. Structurally, this *nido* geometry can be derived from the parent pentagonal bipyramidal *closo* structure by removing one vertex. Specifically, if an additional germanium atom were inserted between Ge1 and Ge3, the resulting seven-vertex cluster would adopt the ideal pentagonal bipyramidal *closo* geometry. This *nido* cluster classification provides a consistent theoretical framework for understanding the bonding characteristics observed in this compound. The electron delocalization within the folded four-membered ring, as evidenced by the alternating bond lengths, is a typical feature of *nido* clusters. Furthermore, the presence of lone pairs on the two unsubstituted germanium atoms (Ge1 and Ge2) is also in good agreement with the electronic requirements of a *nido* Ge_6_ cluster.

### 3.3. Theoretical Description of Metalloid Germanium Cluster ***3***

**Figure 3:**
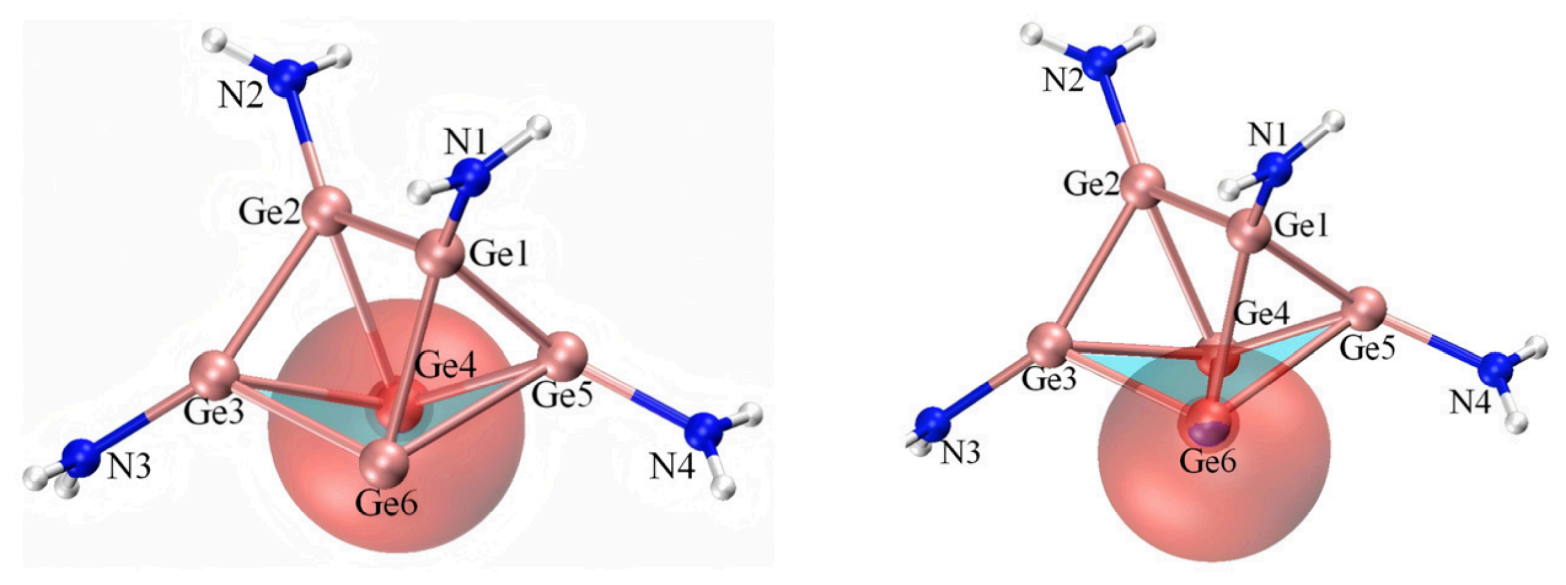
The lone-pair character of the two unsubstituted Ge atoms in the amino-substituted model structure of compound **3**.

To further elucidate the structural characteristics of compound **3**, we made theoretical calculations regarding chemical bonding and molecular orbitals based on its amino-substituted model structure. All DFT calculations were carried out with the TPSS/def2-TZVP level [54,55]. The geometrical parameters obtained from the optimized structures are in good agreement with the experimental data as well as those for isostructural germanium cluster **D**. The two unsubstituted germanium atoms display prominent lone-pair characteristics (Figure 3), suggesting that the steric hindrance differences of the substituents have little influence on the corresponding molecular structure and bonding properties. Partial intrinsic bond orbitals (IBOs) [56] are listed in Figure S10.

### 3.4. NMR and Single-Crystal Structural Characterization of Bis(Amido)-Substituted Germylenes ***4*** and ***6***

To elucidate the structural features and bonding nature of compound **4**, we enhanced the steric bulk of the substituents to explore their effects on the configuration and stability of this type of compound. Treatment of 2 equiv. of KN(Si*^i^*Pr_3_)_2_ **5** with GeCl_2_·dioxane in Et_2_O afforded the bis(amido)-substituted germylene **6**. Owing to the magnetic equivalence of **4**, its ^1^H NMR spectrum shows two singlets at *δ* = 0.42 and 1.07 ppm, which were assigned to the characteristic proton resonances of methyl and tert-butyl groups, respectively. In comparison, the ^1^H NMR spectrum of **6** exhibits a doublet at *δ* = 1.36 ppm attributed to methyl moieties as well as a septet at *δ* = 1.50 ppm arising from the CH protons of isopropyl groups.

**Figure 4:**
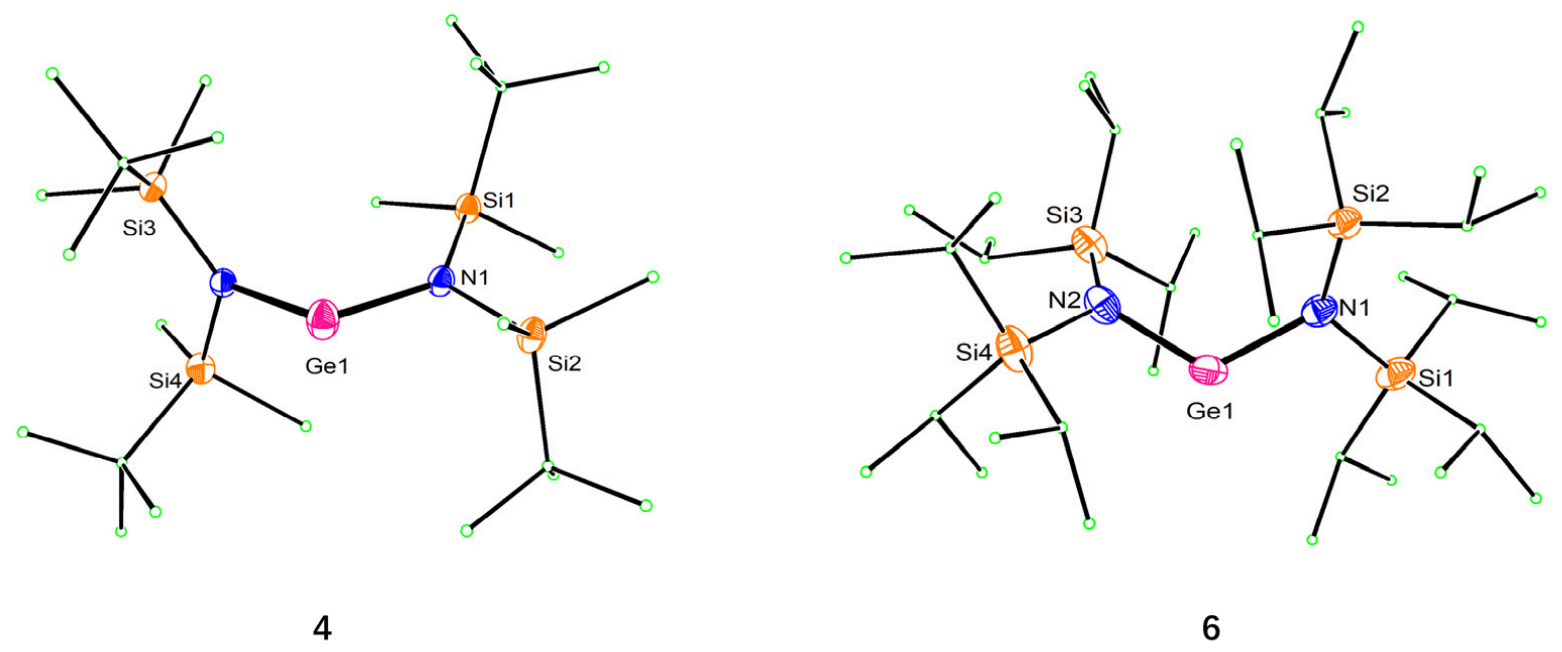
Molecular structure of **4** (**left**) and **6** (**right**). Thermal ellipsoids are drawn at the 50% probability level (except the C atoms of the Me, *^t^*Bu, and *^i^*Pr groups). All hydrogen atoms are omitted for clarity. Selected bond lengths (Å) and angles (°): **4**: Ge1-N1 1.898(3), Ge1-N2 1.925(3), Si1-N1 1.767(3), Si2-N1 1.790(3), Si3-N2 1.756(3), Si4-N2 1.757(2), N1-Ge1-N2 113.54(11), Si1-N1-Si2 121.92(15), Si3-N2-Si4 126.41(16). **6**: Ge1-N1 1.937(2), Ge1-N2 1.931(2), Si1-N1 1.790(2), Si2-N1 1.773(2), Si3-N2 1.763(2), Si4-N2 1.784(2), N1-Ge1-N2 120.58(9), Si1-N1-Si2 125.81(12), Si3-N2-Si4 125.76(12).

The molecular structures of compounds **4** and **6** (Figure 4, left and right) are highly similar, with both adopting a two-coordinate V-shaped geometry at the Ge centers. We propose that the two bulky amido substituents stabilize the divalent oxidation state of the Ge atom via steric and electronic effects. The average Ge-N bond lengths in compound **4** are very close to those in compound **6**, with the former being only 0.022 Å shorter than the latter. Nevertheless, these Ge-N bond lengths are distinctly longer than those in the isostructural analogues [Ge{N(TMS)_2_}_2_] (TMS = SiMe_3_) **F** [57], [Ge(DipNPPh_2_)_2_] (Dip = 2,6-*^i^*Pr_2_C_6_H_3_) **G** [58], and [Ge(Mes_2_AsNPh)_2_] (Mes = 2,4,6-Me_3_C_6_H_2_) **H** [59] (Table 1). The average Si-N bond lengths and Si-N-Si bond angles also exhibit excellent consistency between the two compounds. More importantly, the sum of the bond angles around N1 and N2 in both molecules is close to 360°, which is indicative of an almost trigonal planar geometry at the nitrogen atoms. Notably, a marked difference in the N-Ge-N bond angle was found between compounds **4** and **6**. The N-Ge-N angle of compound **6** (120.58°) is considerably larger than that of compound **4** (113.54°), as well as the angles of **F**, **G,** and **H**. This reveals that the steric bulk of the -Si*^i^*Pr_3_ substituent in **6** is remarkably greater than that of the -Si*^t^*BuMe_2_ moiety in **4** and other amido substituents, leading to a more open N-Ge-N framework in this acyclic amido germylene. These compounds exhibit potential reactivity toward insertion, cycloaddition, and redox reactions, along with good coordination affinity for transition metals and the ability to mediate small-molecule activation.

### 3.5. Theoretical Description of Bis(Amido)-Substituted Germylenes ***4*** and ***6***

**Figure 5:**
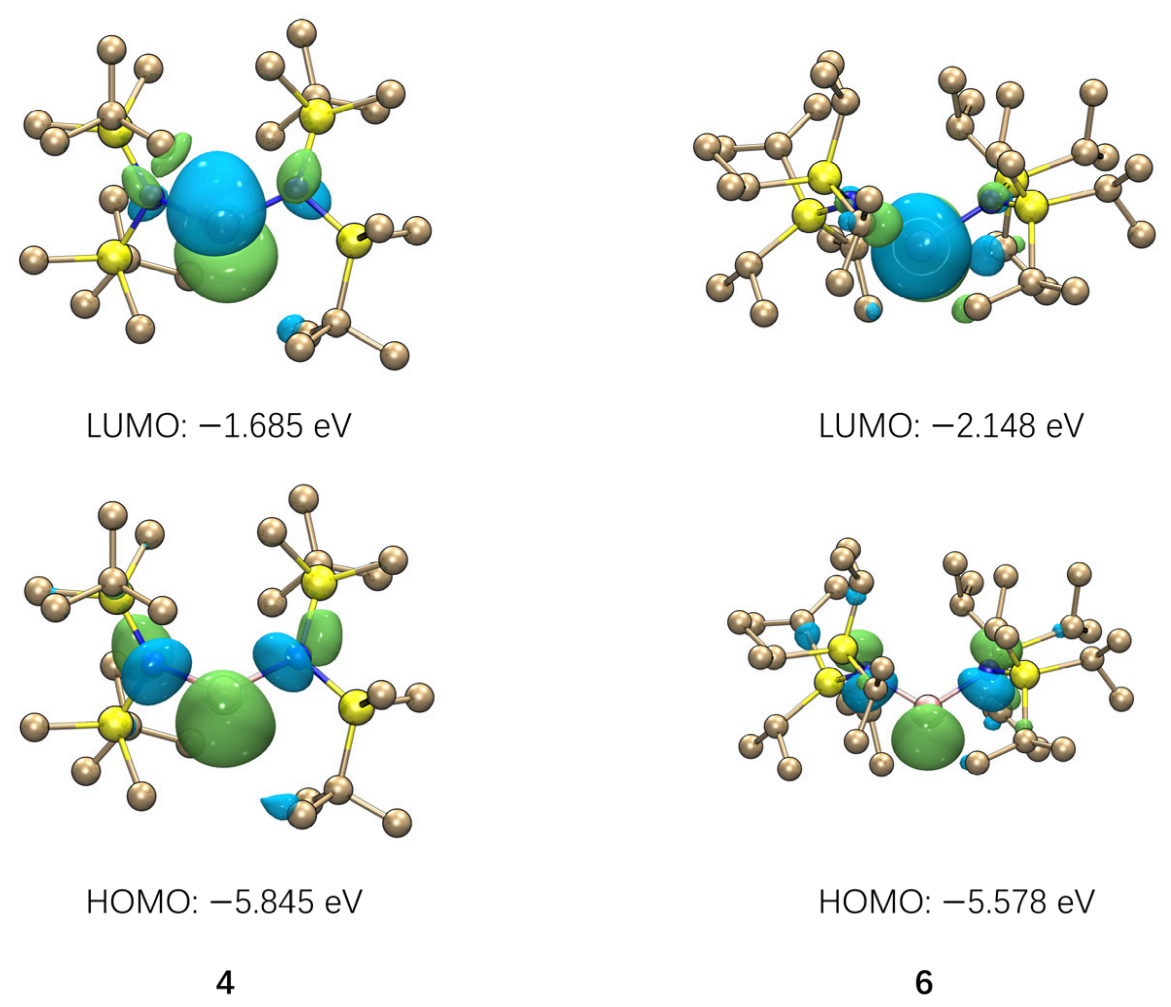
Selected molecular orbitals and their energy states of **4** and **6** at the TPSS/def2-TZVP level.

We further performed theoretical investigations on the chemical bonding and molecular orbitals of compounds **4** and **6** at the same level of theory. The optimized structures of **4** and **6** are in excellent agreement with the single-crystal XRD results. Furthermore, NBO charge analysis was carried out to explore the nature and composition of molecular orbitals. As expected, the energy states of the germylene lone pairs are −5.845 eV for compound **4** and −5.578 eV for compound **6**, indicating that the two compounds exhibit similar *σ*-donor properties (Figure 5). In addition, the LUMO energies of compounds **4** and **6** are −1.685 eV and −2.148 eV, respectively, indicating that compound **6** can function as a stronger *π*-acceptor than **4** in coordination interactions with metal centers.

## 4. Conclusions

In summary, we successfully synthesized and fully characterized a new metalloid Ge_6_ cluster with bulky amide ligands, **3**, along with two bis(amido)-substituted germylenes, **4** and **6**. The Ge_6_ core of **3** adopts a butterfly-shaped geometry capped by a Ge-Ge dumbbell unit, in which the two unsubstituted germanium atoms occupy the apical positions of a distorted square-based pyramid. More importantly, this Ge_6_ cluster can be classified as a *nido* cluster, as its 16 cluster-bonding electrons perfectly satisfy the 2n + 4 electron-counting rule for *nido* clusters. Additionally, the steric hindrance of the amido substituents exerts only a marginal influence on the overall molecular framework of compound **3**. Comparative structural analysis of compounds **4** and **6** reveals that increasing the steric bulk from -Si*^t^*BuMe_2_ to -Si*^i^*Pr_3_ leads to a more open N-Ge-N angle. This finding highlights the decisive role of ligand size in dictating the coordination geometry at Ge^II^ centers. These findings enrich the chemistry surrounding amido-stabilized metalloid Group 14 clusters.

## Figures and Tables

**Table 1:** Selected bond lengths (Å) and angles (°) of compounds **4**, **6** and the reported acyclic bis(amido)-substituted germylene **F**, **G** and **H**.

	4	6	F	G	H
Ge-N (ave.)/Å	1.912(3)	1.934(2)	1.8755(5)	1.8645(3)	1.879(2)
Si-N (ave.)/Å	1.768(3)	1.778(2)			
Si-N-Si/°	124.17(15)	125.79(12)			
N-Ge-N/°	113.54(11)	120.58(9)	107.1(2)	107.1(2)	101.27(13)

## Data Availability

The original contributions presented in this study are included in the article/Supplementary Materials. Further inquiries can be directed to the corresponding authors.

## Supplementary Materials

The following supporting information can be downloaded at: https://www.mdpi.com/article/10.3390/ma19122516/s1, Table S1: Bond lengths [Å] and angles [°] for compound **2**; Table S2: Bond lengths [Å] and angles [°] for compound **3**; Table S3: Bond lengths [Å] and angles [°] for compound **4**; Table S4: Bond lengths [Å] and angles [°] for compound **6**; Figure S1: ^1^H-NMR (400 MHz) spectrum of **2** in C_6_D_6_; Figure S2: ^13^C-NMR (100 MHz) spectrum of **2** in C_6_D_6_; Figure S3: ^29^Si-NMR (600 MHz) spectrum of **2** in C_6_D_6_; Figure S4: ^1^H-NMR (400 MHz) spectrum of **3** in C_6_D_6_; Figure S5: ^13^C-NMR (100 MHz) spectrum of **3** in C_6_D_6_; Figure S6: ^29^Si-NMR (600 MHz) spectrum of **3** in C_6_D_6_; Figure S7: ^1^H-NMR (400 MHz) spectrum of **4** in C_6_D_6_; Figure S8: ^13^C-NMR (100 MHz) spectrum of **4** in C_6_D_6_; Figure S9: ^29^Si-NMR (600 MHz) spectrum of **4** in C_6_D_6_; Figure S10: ^1^H-NMR (400 MHz) spectrum of **6** in C_6_D_6_; Figure S11: ^13^C-NMR (100 MHz) spectrum of **6** in C_6_D_6_; Figure S12: ^29^Si-NMR (600 MHz) spectrum of **6** in C_6_D_6_; Figure S13: Proposed process for the molecular dynamics of **3**. Figure S14: Selected Intrinsic Bond Orbital of **3** at the TPSS/def2-TZVP level; Figure S15: Natural Bond Orbital (NBO) Charge Distributions of Compounds **4** and **6**. References [45,47,54,55,60,61,62] are cited in the supplementary materials.
