# Supplementary material for: Synthesis and Characterization of a Metalloid Ge6 Cluster with Bulky Amide Ligands
Source: Materials (Basel). 2026 Jun 11;19(12):2516. doi: 10.3390/ma19122516 (PMC13304194; doi:10.3390/ma19122516)
Supplement: Supplementary file 1 [file materials-19-02516-s001.zip › materials-4337027-supplementary.pdf]

Supplementary Material for

Synthesis and structural characterization of a metalloid Ge_6 cluster with bulky amide Ligands

Jingjing Liu [1, 2], Xiaoting Liu [1], Bin Zhang [1], Caiting Ji [1], Xiaohui Sun [1], Wenyuan Wang [2]* and Xiaoxu Bo [3*]

[1] *School of Energy Engineering, Shanxi College of Technology, Shuozhou, 036000, China*

[2] *Key Laboratory of Synthetic and Natural Functional Molecule of Ministry of Education, College of Chemistry and Materials Science, Northwest University, Xi'an 710127, China*

[3] *Department of Agriculture and Biotechnology, Wenzhou Vocational College of Science and Technology, Wenzhou 325006, China*

Table of Contents

A. General remarks (2)

B. Crystallographic data for complexes 2, 3, 4 and 6 (3)

C. NMR spectra of 2-4 and 6 (15)

D. Computational details (19)

E. References (20)

A. General remarks

All manipulations were carried out under an atmosphere of purified nitrogen or argon using standard Schlenk lines and glovebox techniques. Glass wares were heatdried and cooled down under vacuum. Hexane was deoxygenated and then refluxed over NaH, another solvents were dried over sodium/benzophenone, distilled and deoxygenated. The solid reactants were weighed in the glove box, and liquid reagents were added with syringe or drip funnel under inert atmosphere. $GeCl_4$ and K were purchased from Arcos. The starting materials $GeCl_2$•dioxane [1], KN(Si^{t}BuMe$_2$)$_2$ [2], and KN(Si^{i}Pr$_3$)$_2$ [3], were prepared according to the literature procedures. Potassium was treated under vigorous stirring and refluxing in hexane under argon, and then cooled rapidly to form potassium grains. The ultra-fine potassium chips was screened and used for the reduction reactions. The solution ^{1}H and ^{13}C{^{1}H} NMR spectra were recorded on JEOL ECZ 400R spectrometer. Chemical shifts of the deuterated solvents in ^{1}H NMR data: C_6D_6, 7.16 ppm, ^{13}C{^{1}H} NMR: C_6D_6, 128.06 ppm. The following abbreviations were used to describe peak patterns when appropriate: s = singlet, d = doublet, t = triplet, m = multiplet, sept = septet. Elemental analysis (C, H, N) was performed with 0.05 mL tin-capsules on a Perkin-Elmer 2400 CHN elemental analyze. Melting points were measured in sealed glass capillaries on TD-V20G melting point apparatus under nitrogen.

The single-crystal X-ray diffractions were performed on Bruker D8 VENTURE PHOTON II detector (compounds **2**, **4**, **6** at 150 K) with a Ga-target Liquid X-ray source (λ = 1.34138 Å). Compound **3** was measured on Bruker D8 Quest detector at 150 K with a Mo-Kα X-ray source (λ = 0.71073 Å). By using Olex2 [4], the structures were solved by direct methods with SHELXT and refined by full-matrix least-squares techniques against F^2 using SHELXL-2014 programs [5]. All thermal displacement parameters were refined anisotropically for non-hydrogen atoms and isotropically for H atoms. The graphical representation of the molecular structures was carried out using Ortep32. Crystal data, details of data collections and refinements can be showed in Table S1-S4.

B. Crystallographic data for complexes 2, 3, 4 and 6

Crystallographic data for compound 2

Identification code	Compound 2
Empirical formula	$C_{12}H_{30}Cl_3GeNSi_2$
Formula weight	423.49
Temperature/K	150.0
Crystal system	triclinic
Space group	P-1
a/Å	9.146(3)
b/Å	9.332(3)
c/Å	13.350(4)
α/°	101.54(2)
β/°	92.173(18)
γ/°	116.609(16)
Volume/Å^3	987.5(6)
Z	2
ρcalcg/cm^3	1.424
μ/mm^{-1}	4.586
F(000)	440.0
Crystal size/mm^3	0.3 × 0.2 × 0.15
Radiation	GaKα (λ = 1.34138)
2Θ range for data collection/°	9.512 to 110.364
Index ranges	$-11 \leq h \leq 11, -11 \leq k \leq 11, -16 \leq l \leq 16$
Reflections collected	13411
Independent reflections	3630 [R_{int} = 0.0494, R_{sigma} = 0.0438]
Data/restraints/parameters	3630/0/182
Goodness-of-fit on F^2	1.093
Final R indexes [I>=2σ (I)]	R_1 = 0.0307, wR_2 = 0.0985
Final R indexes [all data]	R_1 = 0.0327, wR_2 = 0.1009
Largest diff. peak/hole / e Å^{-3}	0.47/-0.60

Table S1. Bond lengths [Å] and angles [°] for compound **2**

Ge1-Cl3	2.1357(9)	Si1-C1	1.899(2)
Ge1-Cl2	2.1333(9)	Si1-C5	1.872(3)
Ge1-Cl1	2.1272(10)	Si1-C6	1.872(2)
Ge1-N1	1.7966(19)	C7-C9	1.544(3)
Si2-N1	1.799(2)	C7-C10	1.533(3)
Si2-C11	1.874(3)	C7-C8	1.535(4)
Si2-C7	1.921(2)	C1-C4	1.538(3)
Si2-C12	1.869(2)	C1-C2	1.537(3)
Si1-N1	1.789(2)	C1-C3	1.538(3)
Cl2-Ge1-Cl3	105.71(4)	C6-Si1-C1	110.91(11)
Cl1-Ge1-Cl3	102.18(4)	Ge1-N1-Si2	115.96(11)

Cl1-Ge1-Cl2	103.94(4)	Si1-N1-Ge1	120.56(10)
N1-Ge1-Cl3	113.69(6)	Si1-N1-Si2	123.48(11)
N1-Ge1-Cl2	112.78(6)	C9-C7-Si2	112.30(16)
N1-Ge1-Cl1	117.24(7)	C10-C7-Si2	110.74(16)
N1-Si2-C11	109.22(10)	C10-C7-C9	108.4(2)
N1-Si2-C7	111.03(9)	C10-C7-C8	107.1(2)
N1-Si2-C12	112.16(11)	C8-C7-Si2	109.68(16)
C11-Si2-C7	111.26(11)	C8-C7-C9	108.5(2)
C12-Si2-C11	101.73(12)	C4-C1-Si1	110.89(16)
C12-Si2-C7	111.09(11)	C4-C1-C3	108.6(2)
N1-Si1-C1	111.05(9)	C2-C1-Si1	112.83(16)
N1-Si1-C5	110.10(11)	C2-C1-C4	108.53(19)
N1-Si1-C6	111.33(10)	C2-C1-C3	107.9(2)
C5-Si1-C1	108.20(11)	C3-C1-Si1	107.94(15)
C5-Si1-C6	105.03(12)		

Crystallographic data for compound 3

Identification code	Compound 3
Empirical formula	$C_{48}H_{120}Ge_6N_4Si_8$
Formula weight	1413.73
Temperature/K	150.0
Crystal system	triclinic
Space group	P-1
a/Å	13.0104(14)
b/Å	14.2441(16)
c/Å	20.254(2)
α/°	86.907(4)
β/°	86.071(4)
γ/°	74.033(3)
Volume/Å^3	3597.9(7)
Z	2
ρcalcg/cm^3	1.305
μ/mm^{-1}	2.639
F(000)	1480.0
Crystal size/mm^3	0.2 × 0.1 × 0.1
Radiation	MoKα (λ = 0.71073)
2Θ range for data collection/°	2.016 to 51.408
Index ranges	$-15 \le h \le 15$, $-17 \le k \le 17$, $-24 \le l \le 24$

Reflections collected	65654
Independent reflections	13627 [R_{int} = 0.0649, R_{sigma} = 0.0464]
Data/restraints/parameters	13627/3/617
Goodness-of-fit on F^2	1.053
Final R indexes [I>=2σ (I)]	R_1 = 0.0422, wR_2 = 0.1204
Final R indexes [all data]	R_1 = 0.0493, wR_2 = 0.1259
Largest diff. peak/hole / e Å^{-3}	1.48/-0.97

Table S2. Bond lengths [Å] and angles [°] for compound **3**

Ge1-Ge2	2.6026(6)	Si5-C25	1.872(4)
Ge1-Ge5	2.4824(5)	Si5-C27	1.932(4)
Ge1-Ge6	2.6163(6)	Si5-C26	1.872(4)
Ge1-N1	1.897(3)	Si7-N4	1.761(3)
Ge2-Ge3	2.4914(5)	Si7-C45	1.904(4)
Ge2-Ge4	2.6162(6)	Si7-C43	1.873(5)
Ge2-N2	1.901(3)	Si7-C44	1.871(4)
Ge3-Ge4	2.7439(6)	Si8-N4	1.763(3)
Ge3-Ge6	2.4654(6)	Si8-C38	1.875(5)
Ge3-N3	1.892(2)	Si8-C37	1.887(5)
Ge5-Ge4	2.4676(6)	Si8-C39	1.914(7)
Ge5-Ge6	2.7368(6)	C15-C17	1.520(5)
Ge5-N4	1.897(3)	C15-C18	1.538(5)
Ge4-Ge6	2.6820(6)	C15-C16	1.541(5)
Si3-N2	1.763(3)	C3-C6	1.529(5)
Si3-C15	1.905(4)	C3-C4	1.521(7)
Si3-C13	1.872(4)	C3-C5	1.538(5)
Si3-C14	1.869(3)	C9-C11	1.541(5)
Si4-N2	1.768(3)	C9-C12	1.537(6)
Si4-C20	1.867(4)	C9-C10	1.532(5)
Si4-C19	1.879(4)	C45-C48	1.533(5)
Si4-C21	1.906(4)	C45-C46	1.534(6)
Si2-N1	1.768(3)	C45-C47	1.546(5)
Si2-C8	1.869(3)	C33-C36	1.540(5)
Si2-C9	1.911(4)	C33-C35	1.542(5)
Si2-C7	1.878(4)	C33-C34	1.533(5)
Si6-N3	1.765(3)	C21-C24	1.523(6)
Si6-C31	1.871(4)	C21-C22	1.537(5)
Si6-C33	1.906(4)	C21-C23	1.526(5)
Si6-C32	1.884(4)	C27-C30	1.526(6)
Si1-N1	1.765(3)	C27-C29	1.535(5)

Si1-C3	1.904(4)	C27-C28	1.539(6)
Si1-C1	1.868(4)	C39-C40	1.466(13)
Si1-C2	1.876(4)	C39-C42	1.523(11)
Si5-N3	1.764(3)	C39-C41	1.572(14)
Ge2-Ge1-Ge6	85.534(16)	N4-Si7-C45	112.65(16)
Ge5-Ge1-Ge2	81.982(16)	N4-Si7-C43	109.75(17)
Ge5-Ge1-Ge6	64.864(17)	N4-Si7-C44	112.9(2)
N1-Ge1-Ge2	136.43(8)	C43-Si7-C45	108.0(2)
N1-Ge1-Ge5	132.28(8)	C44-Si7-C45	107.23(19)
N1-Ge1-Ge6	129.67(8)	C44-Si7-C43	106.0(3)
Ge1-Ge2-Ge4	85.638(16)	N4-Si8-C38	109.35(17)
Ge3-Ge2-Ge1	82.001(16)	N4-Si8-C37	112.56(18)
Ge3-Ge2-Ge4	64.936(16)	N4-Si8-C39	113.1(4)
N2-Ge2-Ge1	135.54(8)	C38-Si8-C37	104.3(3)
N2-Ge2-Ge3	132.52(7)	C38-Si8-C39	108.3(3)
N2-Ge2-Ge4	130.52(8)	C37-Si8-C39	108.8(3)
Ge2-Ge3-Ge4	59.730(15)	Si6-N3-Ge3	115.07(14)
Ge6-Ge3-Ge2	91.273(16)	Si5-N3-Ge3	119.51(14)
Ge6-Ge3-Ge4	61.698(15)	Si5-N3-Si6	124.97(14)
N3-Ge3-Ge2	142.37(8)	Si3-N2-Ge2	118.34(14)
N3-Ge3-Ge4	118.80(8)	Si3-N2-Si4	124.87(15)
N3-Ge3-Ge6	122.33(8)	Si4-N2-Ge2	116.65(13)
Ge1-Ge5-Ge6	59.936(15)	Si2-N1-Ge1	117.08(14)
Ge4-Ge5-Ge1	91.549(17)	Si1-N1-Ge1	119.07(14)
Ge4-Ge5-Ge6	61.783(15)	Si1-N1-Si2	123.82(15)
N4-Ge5-Ge1	140.71(9)	Si7-N4-Ge5	115.08(16)
N4-Ge5-Ge4	123.68(9)	Si7-N4-Si8	125.76(16)
N4-Ge5-Ge6	118.62(8)	Si8-N4-Ge5	119.07(15)
Ge2-Ge4-Ge3	55.334(13)	C17-C15-Si3	112.7(2)
Ge2-Ge4-Ge6	83.952(16)	C17-C15-C18	109.1(3)
Ge5-Ge4-Ge2	81.987(17)	C17-C15-C16	107.0(3)
Ge5-Ge4-Ge3	104.917(18)	C18-C15-Si3	109.8(3)
Ge5-Ge4-Ge6	64.050(16)	C18-C15-C16	108.8(3)
Ge6-Ge4-Ge3	54.036(15)	C16-C15-Si3	109.2(3)
Ge1-Ge6-Ge5	55.200(13)	C6-C3-Si1	111.2(2)
Ge1-Ge6-Ge4	84.047(16)	C6-C3-C4	109.2(3)
Ge3-Ge6-Ge1	82.215(16)	C6-C3-C5	108.3(3)
Ge3-Ge6-Ge5	105.190(17)	C4-C3-Si1	111.0(2)
Ge3-Ge6-Ge4	64.266(16)	C4-C3-C5	107.5(3)
Ge4-Ge6-Ge5	54.168(15)	C5-C3-Si1	109.5(3)
N2-Si3-C15	113.69(14)	C11-C9-Si2	108.8(3)

N2-Si3-C13	112.71(15)	C12-C9-Si2	110.4(3)
N2-Si3-C14	109.86(15)	C12-C9-C11	108.2(3)
C13-Si3-C15	104.77(17)	C10-C9-Si2	112.6(3)
C14-Si3-C15	110.05(17)	C10-C9-C11	108.5(3)
C14-Si3-C13	105.35(18)	C10-C9-C12	108.2(4)
N2-Si4-C20	109.01(14)	C48-C45-Si7	110.2(2)
N2-Si4-C19	112.21(15)	C48-C45-C46	108.9(4)
N2-Si4-C21	114.18(14)	C48-C45-C47	109.0(3)
C20-Si4-C19	105.20(18)	C46-C45-Si7	110.5(3)
C20-Si4-C21	107.26(17)	C46-C45-C47	108.5(4)
C19-Si4-C21	108.48(17)	C47-C45-Si7	109.6(3)
N1-Si2-C8	109.12(14)	C36-C33-Si6	110.6(2)
N1-Si2-C9	114.74(15)	C36-C33-C35	107.5(3)
N1-Si2-C7	111.64(15)	C35-C33-Si6	109.6(3)
C8-Si2-C9	106.56(16)	C34-C33-Si6	111.7(3)
C8-Si2-C7	105.24(18)	C34-C33-C36	107.7(3)
C7-Si2-C9	109.00(19)	C34-C33-C35	109.7(3)
N3-Si6-C31	109.81(15)	C24-C21-Si4	109.7(3)
N3-Si6-C33	112.14(14)	C24-C21-C22	107.9(3)
N3-Si6-C32	112.06(17)	C24-C21-C23	108.1(4)
C31-Si6-C33	108.05(18)	C22-C21-Si4	109.6(3)
C31-Si6-C32	105.88(19)	C23-C21-Si4	112.9(3)
C32-Si6-C33	108.65(18)	C23-C21-C22	108.4(4)
N1-Si1-C3	113.59(14)	C30-C27-Si5	112.3(3)
N1-Si1-C1	109.65(16)	C30-C27-C29	108.8(4)
N1-Si1-C2	113.97(15)	C30-C27-C28	107.0(4)
C1-Si1-C3	109.36(18)	C29-C27-Si5	111.0(3)
C1-Si1-C2	104.9(2)	C29-C27-C28	107.2(4)
C2-Si1-C3	104.94(18)	C28-C27-Si5	110.3(3)
N3-Si5-C25	108.44(15)	C40-C39-Si8	114.0(5)
N3-Si5-C27	113.04(16)	C40-C39-C42	115.8(9)
N3-Si5-C26	112.51(16)	C40-C39-C41	105.0(7)
C25-Si5-C27	110.06(18)	C42-C39-Si8	112.7(6)
C25-Si5-C26	103.4(2)	C42-C39-C41	102.0(7)
C26-Si5-C27	108.93(19)	C41-C39-Si8	105.6(7)

Crystallographic data for compound 4

Identification code	Compound 4
Empirical formula	$C_{24}H_{60}GeN_2Si_4$

Formula weight	561.69
Temperature/K	150.15
Crystal system	orthorhombic
Space group	$P2_12_12_1$
a/Å	11.7939(9)
b/Å	14.1145(13)
c/Å	19.656(5)
α/°	90
β/°	90
γ/°	90
Volume/Å^3	3272.1(9)
Z	4
ρcalcg/cm^3	1.140
μ/mm^{-1}	1.847
F(000)	1224.0
Crystal size/mm^3	0.3 × 0.15 × 0.1
Radiation	GaKα (λ = 1.34138)
2Θ range for data collection/°	6.708 to 109.954
Index ranges	-14 ≤ h ≤ 14, -15 ≤ k ≤ 17, -23 ≤ l ≤ 23
Reflections collected	21278
Independent reflections	6073 [R_{int} = 0.0617, R_{sigma} = 0.0554]
Data/restraints/parameters	6073/0/300
Goodness-of-fit on F^2	1.096
Final R indexes [I>=2σ (I)]	R_1 = 0.0340, wR_2 = 0.0898
Final R indexes [all data]	R_1 = 0.0387, wR_2 = 0.0928
Largest diff. peak/hole / e Å^{-3}	0.57/-0.66

Table S3. Bond lengths [Å] and angles [°] for compound **4**

Ge1-N2	1.925(3)	Si2-C7	1.908(3)
Ge1-N1	1.898(3)	Si2-C11	1.872(4)
Si3-N2	1.756(3)	Si2-C12	1.871(4)
Si3-C13	1.919(3)	C19-C21	1.547(5)
Si3-C17	1.886(4)	C19-C20	1.540(6)
Si3-C18	1.866(4)	C19-C22	1.542(5)
Si4-N2	1.757(2)	C13-C16	1.534(5)
Si4-C19	1.913(4)	C13-C14	1.528(5)
Si4-C23	1.881(4)	C13-C15	1.544(5)
Si4-C24	1.870(3)	C1-C2	1.537(5)
Si1-N1	1.767(3)	C1-C3	1.537(5)

Si1-C5	1.871(4)	C1-C4	1.548(5)
Si1-C1	1.940(3)	C8-C7	1.545(6)
Si1-C6	1.882(3)	C7-C9	1.539(6)
Si2-N1	1.790(3)	C7-C10	1.546(6)
N1-Ge1-N2	113.54(11)	Si1-N1-Ge1	131.93(15)
N2-Si3-C13	114.93(14)	Si1-N1-Si2	121.92(15)
N2-Si3-C17	108.46(14)	Si2-N1-Ge1	106.01(13)
N2-Si3-C18	112.64(15)	C21-C19-Si4	109.5(3)
C17-Si3-C13	104.17(15)	C20-C19-Si4	112.8(3)
C18-Si3-C13	109.58(17)	C20-C19-C21	106.3(3)
C18-Si3-C17	106.36(18)	C20-C19-C22	110.0(4)
N2-Si4-C19	112.64(14)	C22-C19-Si4	110.7(3)
N2-Si4-C23	113.04(15)	C22-C19-C21	107.4(3)
N2-Si4-C24	112.34(15)	C16-C13-Si3	111.1(2)
C23-Si4-C19	107.54(18)	C16-C13-C15	108.2(3)
C24-Si4-C19	106.48(18)	C14-C13-Si3	113.9(2)
C24-Si4-C23	104.22(19)	C14-C13-C16	107.9(3)
N1-Si1-C5	111.56(14)	C14-C13-C15	107.4(3)
N1-Si1-C1	111.96(14)	C15-C13-Si3	108.1(3)
N1-Si1-C6	112.33(14)	C2-C1-Si1	111.5(2)
C5-Si1-C1	109.51(14)	C2-C1-C3	107.8(3)
C5 Si1 C6	102.54(17)	C2-C1-C4	108.9(3)
C6-Si1-C1	108.50(16)	C3-C1-Si1	110.3(2)
N1-Si2-C7	112.62(16)	C3-C1-C4	105.7(3)
N1-Si2-C11	109.98(15)	C4-C1-Si1	112.4(2)
N1-Si2-C12	114.40(15)	C8-C7-Si2	111.8(2)
C11-Si2-C7	109.17(18)	C8-C7-C10	108.4(3)
C12-Si2-C7	107.14(18)	C9-C7-Si2	111.6(3)
C12-Si2-C11	103.0(2)	C9-C7-C8	109.2(4)
Si3-N2-Ge1	110.47(12)	C9-C7-C10	107.2(3)
Si3-N2-Si4	126.41(16)	C10-C7-Si2	108.5(3)
Si4-N2-Ge1	121.18(15)		

Crystallographic data for compound 6

Identification code	Compound 6
Empirical formula	$C_{36}H_{84}GeN_2Si_4$
Formula weight	730.00
Temperature/K	150.00
Crystal system	triclinic

Space group	P-1
a/Å	17.724(5)
b/Å	20.708(5)
c/Å	21.738(5)
α/°	115.674(9)
β/°	111.580(13)
γ/°	91.512(14)
Volume/Å^3	6520(3)
Z	6
ρcalcg/cm^3	1.115
μ/mm^{-1}	1.447
F(000)	2412.0
Crystal size/mm^3	0.05 × 0.03 × 0.02
Radiation	GaKα (λ = 1.34138)
2Θ range for data collection/°	4.224 to 110.948
Index ranges	$-21 \le h \le 21$, $-25 \le k \le 25$, $-26 \le l \le 26$
Reflections collected	136366
Independent reflections	24974 [R_{int} = 0.0710, R_{sigma} = 0.0477]
Data/restraints/parameters	24974/166/1428
Goodness-of-fit on F^2	1.075
Final R indexes [I>=2σ (I)]	R_1 = 0.0484, wR_2 = 0.1277
Final R indexes [all data]	R_1 = 0.0615, wR_2 = 0.1365
Largest diff. peak/hole / e Å^{-3}	1.84/-1.61

Table S4. Bond lengths [Å] and angles [°] for compound **6**

Ge3-N5	1.941(2)	C16-C18	1.540(3)
Ge3-N6	1.950(2)	C31-C33	1.521(5)
Ge1-N1	1.937(2)	C31-C32	1.548(4)
Ge1-N2	1.931(2)	C28-C29	1.533(4)
Ge2-N3	1.968(2)	C28-C30	1.539(4)
Ge2-N4	1.918(2)	C34-C36	1.531(5)
Si8-N4	1.794(2)	C34-C35	1.542(4)
Si8-C67	1.898(3)	C25-C26	1.551(4)
Si8-C70	1.910(3)	C25-C27	1.533(4)
Si8-C64	1.902(3)	C19-C20	1.541(4)
Si7-N4	1.769(2)	C19-C21	1.542(5)
Si7-C58	1.881(3)	C22-C23	1.530(4)
Si7-C55	1.902(3)	C22-C24	1.527(4)
Si7-C61	1.882(3)	C73-C74	1.538(4)

Si1-N1	1.790(2)	C73-C75	1.532(5)
Si1-C7	1.891(3)	C76-C77	1.532(4)
Si1-C4	1.904(3)	C76-C78	1.536(4)
Si1-C1	1.906(3)	C79-C80	1.542(4)
Si2-N1	1.773(2)	C79-C81	1.525(4)
Si2-C13	1.911(3)	C82-C83	1.536(4)
Si2-C10	1.887(3)	C82-C84	1.540(4)
Si2-C16	1.914(3)	C85-C86	1.531(3)
Si4-N2	1.784(2)	C85-C87	1.534(3)
Si4-C31	1.906(3)	C88-C89	1.532(4)
Si4-C28	1.906(3)	C88-C90	1.539(3)
Si4-C34	1.903(3)	C91-C92	1.536(4)
Si3-N2	1.763(2)	C91-C93	1.534(4)
Si3-C25	1.909(3)	C94-C95	1.531(4)
Si3-C19	1.911(3)	C94-C96	1.522(4)
Si3-C22	1.902(3)	C97-C98	1.524(4)
Si9-N5	1.774(2)	C97-C99	1.542(4)
Si9-C73	1.912(3)	C100-C101	1.536(4)
Si9-C76	1.904(3)	C100-C102	1.541(4)
Si9-C79	1.910(3)	C103-C104	1.541(4)
Si10-N5	1.769(2)	C103-C105	1.520(4)
Si10-C82	1.915(3)	C106-C107	1.550(4)
Si10-C85	1.899(2)	C106-C108	1.522(4)
Si10-C88	1.892(2)	Si5-C43	1.901(6)
Si11-N6	1.772(2)	Si5-C40	1.907(6)
Si11-C91	1.917(3)	Si5-C37	1.892(8)
Si11-C94	1.898(3)	C43-C44	1.533(7)
Si11-C97	1.914(3)	C43-C45	1.546(7)
Si12-N6	1.772(2)	C40-C41	1.532(7)
Si12-C100	1.899(3)	C40-C42	1.545(8)
Si12-C103	1.898(3)	C37-C39	1.551(7)
Si12-C106	1.918(3)	C37-C38	1.532(8)
N3-Si5	1.764(2)	Si1'-C1'	1.904(7)
N3-Si1'	1.822(3)	Si1'-C4'	1.910(6)
N3-Si6	1.783(3)	Si1'-C7'	1.888(5)
N3-Si2'	1.704(4)	C1'-C2'	1.533(7)
C67-C68	1.528(4)	C1'-C3'	1.519(7)
C67-C69	1.545(4)	C4'-C5'	1.531(7)
C70-C71	1.524(4)	C4'-C6'	1.540(7)
C70-C72	1.529(4)	C7'-C8'	1.546(7)
C64-C65	1.538(4)	C7'-C9'	1.520(7)

C64-C66	1.539(4)	Si6-C49	1.904(7)
C58-C59	1.528(4)	Si6-C46	1.907(8)
C58-C60	1.534(4)	Si6-C52	1.901(5)
C55-C57	1.517(5)	C49-C51	1.540(8)
C55-C56	1.540(4)	C49-C50	1.540(7)
C61-C63	1.555(5)	C46-C48	1.536(8)
C61-C62	1.434(6)	C46-C47	1.528(8)
C7-C9	1.545(4)	C52-C53	1.520(7)
C7-C8	1.535(4)	C52-C54	1.536(6)
C4-C5	1.540(4)	Si2'-C10'	1.900(7)
C4-C6	1.531(4)	Si2'-C13'	1.973(6)
C1-C2	1.545(4)	Si2'-C16'	1.898(6)
C1-C3	1.540(4)	C10'-C11'	1.536(9)
C13-C15	1.540(4)	C10'-C12'	1.536(8)
C13-C14	1.540(4)	C13'-C14'	1.517(8)
C10-C12	1.529(4)	C13'-C15'	1.535(7)
C10-C11	1.539(4)	C16'-C17'	1.527(7)
C16-C17	1.522(4)	C16'-C18'	1.540(7)
N5-Ge3-N6	120.55(8)	C17-C16-Si2	114.85(18)
N2-Ge1-N1	120.58(9)	C17-C16-C18	110.3(2)
N4-Ge2-N3	121.43(9)	C18-C16-Si2	116.90(18)
N4-Si8-C67	110.84(11)	C33-C31-Si4	118.1(2)
N4-Si8-C70	105.95(11)	C33-C31-C32	107.6(3)
N4-Si8-C64	113.81(11)	C32-C31-Si4	113.0(2)
C67-Si8-C70	109.27(12)	C29-C28-Si4	115.9(2)
C67-Si8-C64	108.07(12)	C29-C28-C30	108.9(3)
C64-Si8-C70	108.81(12)	C30-C28-Si4	117.8(2)
N4-Si7-C58	108.35(11)	C36-C34-Si4	113.5(2)
N4-Si7-C55	105.56(15)	C36-C34-C35	109.1(3)
N4-Si7-C61	114.67(13)	C35-C34-Si4	113.8(2)
C58-Si7-C55	106.68(14)	C26-C25-Si3	117.1(2)
C58-Si7-C61	113.17(16)	C27-C25-Si3	115.0(2)
C61-Si7-C55	107.8(2)	C27-C25-C26	109.3(2)
N1-Si1-C7	111.48(11)	C20-C19-Si3	112.7(2)
N1-Si1-C4	107.04(11)	C20-C19-C21	108.6(3)
N1-Si1-C1	111.37(12)	C21-C19-Si3	117.0(2)
C7-Si1-C4	108.64(13)	C23-C22-Si3	115.2(2)
C7-Si1-C1	108.64(13)	C24-C22-Si3	115.5(2)
C4-Si1-C1	109.63(14)	C24-C22-C23	109.4(3)
N1-Si2-C13	114.49(11)	C74-C73-Si9	112.8(2)
N1-Si2-C10	111.30(10)	C75-C73-Si9	115.0(2)

N1-Si2-C16	105.04(10)	C75-C73-C74	109.0(3)
C13-Si2-C16	108.61(12)	C77-C76-Si9	116.6(2)
C10-Si2-C13	110.71(12)	C77-C76-C78	109.7(2)
C10-Si2-C16	106.16(11)	C78-C76-Si9	115.69(19)
N2-Si4-C31	109.81(12)	C80-C79-Si9	114.8(2)
N2-Si4-C28	106.48(12)	C81-C79-Si9	115.4(2)
N2-Si4-C34	112.97(12)	C81-C79-C80	107.8(3)
C28-Si4-C31	110.93(14)	C83-C82-Si10	116.60(18)
C34-Si4-C31	107.62(14)	C83-C82-C84	108.2(2)
C34-Si4-C28	109.06(14)	C84-C82-Si10	112.78(18)
N2-Si3-C25	104.20(12)	C86-C85-Si10	115.38(18)
N2-Si3-C19	113.72(12)	C86-C85-C87	109.2(2)
N2-Si3-C22	112.02(12)	C87-C85-Si10	117.59(17)
C25-Si3-C19	108.73(13)	C89-C88-Si10	114.46(17)
C22-Si3-C25	106.45(13)	C89-C88-C90	108.6(2)
C22-Si3-C19	111.17(14)	C90-C88-Si10	116.17(18)
N5-Si9-C73	110.26(11)	C92-C91-Si11	116.8(2)
N5-Si9-C76	106.88(11)	C93-C91-Si11	114.3(2)
N5-Si9-C79	112.22(11)	C93-C91-C92	107.5(2)
C76-Si9-C73	109.89(13)	C95-C94-Si11	115.3(2)
C76-Si9-C79	108.24(13)	C96-C94-Si11	117.9(2)
C79-Si9-C73	109.29(14)	C96-C94-C95	109.8(2)
N5-Si10-C82	115.21(10)	C98-C97-Si11	114.7(2)
N5-Si10-C85	104.49(10)	C98-C97-C99	108.1(2)
N5-Si10-C88	109.53(10)	C99-C97-Si11	113.0(2)
C85-Si10-C82	109.23(11)	C101-C100-Si12	114.63(18)
C88-Si10-C82	110.63(11)	C101-C100-C102	109.0(2)
C88-Si10-C85	107.30(11)	C102-C100-Si12	117.8(2)
N6-Si11-C91	109.54(11)	C104-C103-Si12	114.5(2)
N6-Si11-C94	105.90(11)	C105-C103-Si12	116.27(18)
N6-Si11-C97	113.30(11)	C105-C103-C104	109.1(2)
C94-Si11-C91	111.21(13)	C107-C106-Si12	113.86(19)
C94-Si11-C97	108.77(13)	C108-C106-Si12	116.2(2)
C97-Si11-C91	108.15(13)	C108-C106-C107	107.4(2)
N6-Si12-C100	103.92(11)	N3-Si5-C43	104.3(2)
N6-Si12-C103	112.71(11)	N3-Si5-C40	107.8(2)
N6-Si12-C106	114.29(11)	N3-Si5-C37	115.5(3)
C100-Si12-C106	108.23(12)	C43-Si5-C40	112.7(3)
C103-Si12-C100	106.98(12)	C37-Si5-C43	107.3(4)
C103-Si12-C106	110.17(13)	C37-Si5-C40	109.2(4)
Si5-N3-Ge2	105.02(11)	C44-C43-Si5	115.7(5)

Si5-N3-Si6	125.39(15)	C44-C43-C45	109.7(6)
Si1'-N3-Ge2	115.02(11)	C45-C43-Si5	117.4(5)
Si6-N3-Ge2	120.76(14)	C41-C40-Si5	113.9(5)
Si2'-N3-Ge2	110.84(15)	C41-C40-C42	108.0(6)
Si2'-N3-Si1'	129.74(17)	C42-C40-Si5	117.4(5)
Si8-N4-Ge2	110.09(10)	C39-C37-Si5	113.6(6)
Si7-N4-Ge2	124.41(11)	C38-C37-Si5	114.7(6)
Si7-N4-Si8	125.23(11)	C38-C37-C39	109.2(7)
Si1-N1-Ge1	109.34(10)	N3-Si1'-C1'	119.4(3)
Si2-N1-Ge1	122.04(11)	N3-Si1'-C4'	107.7(2)
Si2-N1-Si1	125.81(12)	N3-Si1'-C7'	112.1(2)
Si4-N2-Ge1	110.68(11)	C1'-Si1'-C4'	104.7(3)
Si3-N2-Ge1	119.75(11)	C7'-Si1'-C1'	103.5(3)
Si3-N2-Si4	125.76(12)	C7'-Si1'-C4'	108.8(3)
Si9-N5-Ge3	110.08(10)	C2'-C1'-Si1'	115.0(5)
Si10-N5-Ge3	123.18(11)	C3'-C1'-Si1'	118.0(5)
Si10-N5-Si9	125.30(11)	C3'-C1'-C2'	108.3(5)
Si11-N6-Ge3	110.28(10)	C5'-C4'-Si1'	116.3(4)
Si12 N6 Ge3	118.83(11)	C5'-C4'-C6'	108.2(5)
Si12-N6-Si11	125.67(12)	C6'-C4'-Si1'	115.9(5)
C68-C67-Si8	115.0(2)	C8'-C7'-Si1'	113.0(4)
C68-C67-C69	108.6(2)	C9'-C7'-Si1'	115.8(6)
C69-C67-Si8	113.4(2)	C9'-C7'-C8'	108.4(6)
C71-C70-Si8	112.87(18)	N3-Si6-C49	116.0(3)
C71-C70-C72	110.3(2)	N3-Si6-C46	109.9(3)
C72-C70-Si8	118.2(2)	N3-Si6-C52	106.2(2)
C65-C64-Si8	114.61(19)	C49-Si6-C46	108.6(5)
C65-C64-C66	108.2(2)	C52-Si6-C49	110.0(3)
C66-C64-Si8	115.1(2)	C52-Si6-C46	105.7(4)
C59-C58-Si7	116.6(2)	C51-C49-Si6	118.5(9)
C59-C58-C60	108.3(2)	C51-C49-C50	108.2(7)
C60-C58-Si7	114.70(18)	C50-C49-Si6	112.8(5)
C57-C55-Si7	118.8(3)	C48-C46-Si6	115.3(6)
C57-C55-C56	109.1(3)	C47-C46-Si6	116.7(6)
C56-C55-Si7	114.9(2)	C47-C46-C48	108.5(7)
C63-C61-Si7	111.2(3)	C53-C52-Si6	114.7(4)
C62-C61-Si7	118.4(3)	C53-C52-C54	110.8(5)
C62-C61-C63	108.3(4)	C54-C52-Si6	117.8(4)
C9-C7-Si1	115.7(2)	N3-Si2'-C10'	116.6(4)
C8-C7-Si1	113.2(2)	N3-Si2'-C13'	112.3(2)
C8-C7-C9	108.9(2)	N3-Si2'-C16'	101.2(3)

C5-C4-Si1	117.1(2)	C10'-Si2'-C13'	109.4(4)
C6-C4-Si1	115.4(2)	C16'-Si2'-C10'	108.9(4)
C6-C4-C5	109.5(3)	C16'-Si2'-C13'	107.9(3)
C2-C1-Si1	112.8(2)	C11'-C10'-Si2'	114.4(9)
C3-C1-Si1	117.6(2)	C12'-C10'-Si2'	112.7(6)
C3-C1-C2	107.2(3)	C12'-C10'-C11'	108.5(9)
C15-C13-Si2	117.1(2)	C14'-C13'-Si2'	111.6(6)
C15-C13-C14	108.2(2)	C14'-C13'-C15'	111.0(7)
C14-C13-Si2	112.20(19)	C15'-C13'-Si2'	113.9(5)
C12-C10-Si2	114.60(17)	C17'-C16'-Si2'	113.9(4)
C12-C10-C11	108.7(2)	C17'-C16'-C18'	109.1(6)
C11-C10-Si2	116.65(19)	C18'-C16'-Si2'	119.3(5)

C. NMR spectra of 2-4 and 6

Compound 2

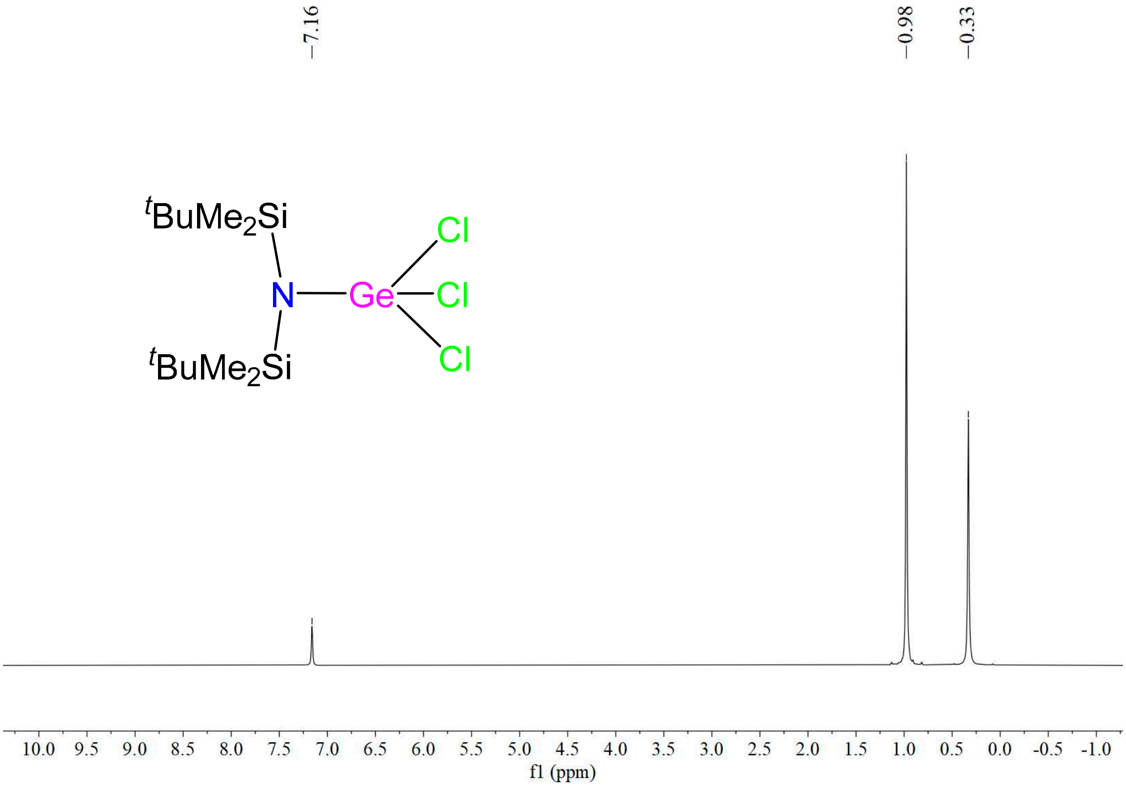

Figure S1. ^{1}H-NMR (400 MHz) spectrum of **2** in C_6D_6.

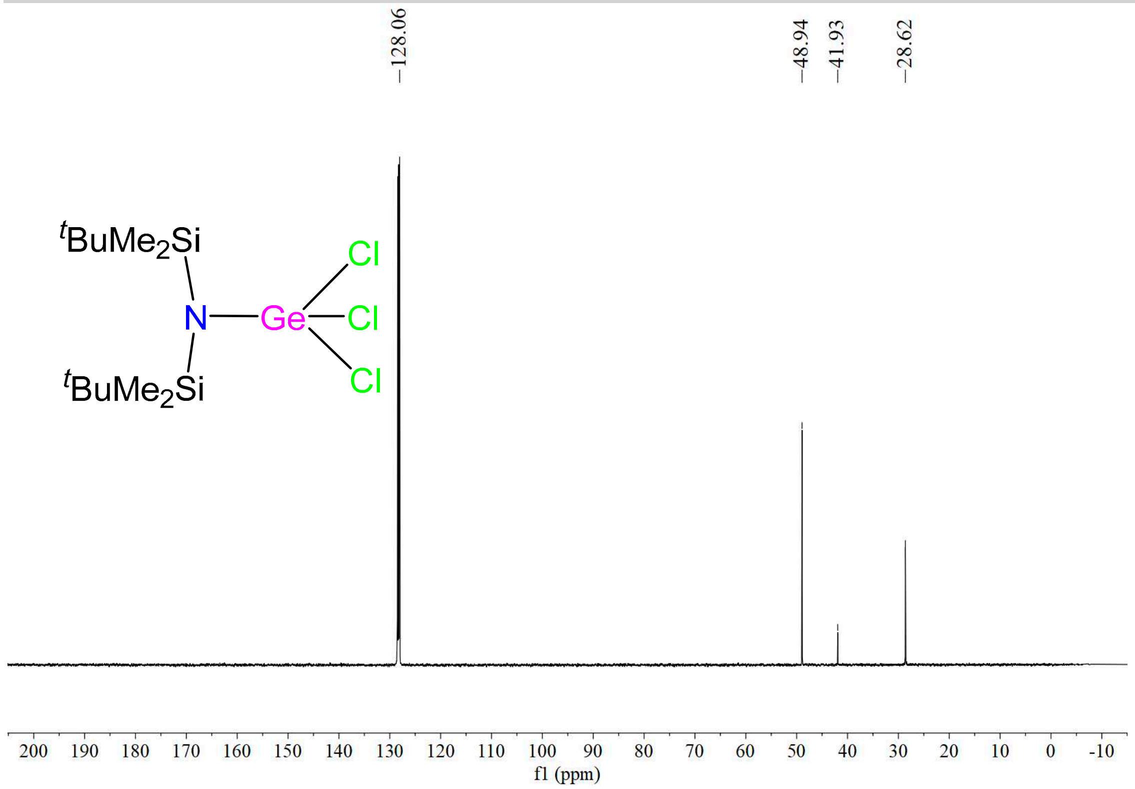

Figure S2. ^{13}C-NMR (100 MHz) spectrum of **2** in C_6D_6.

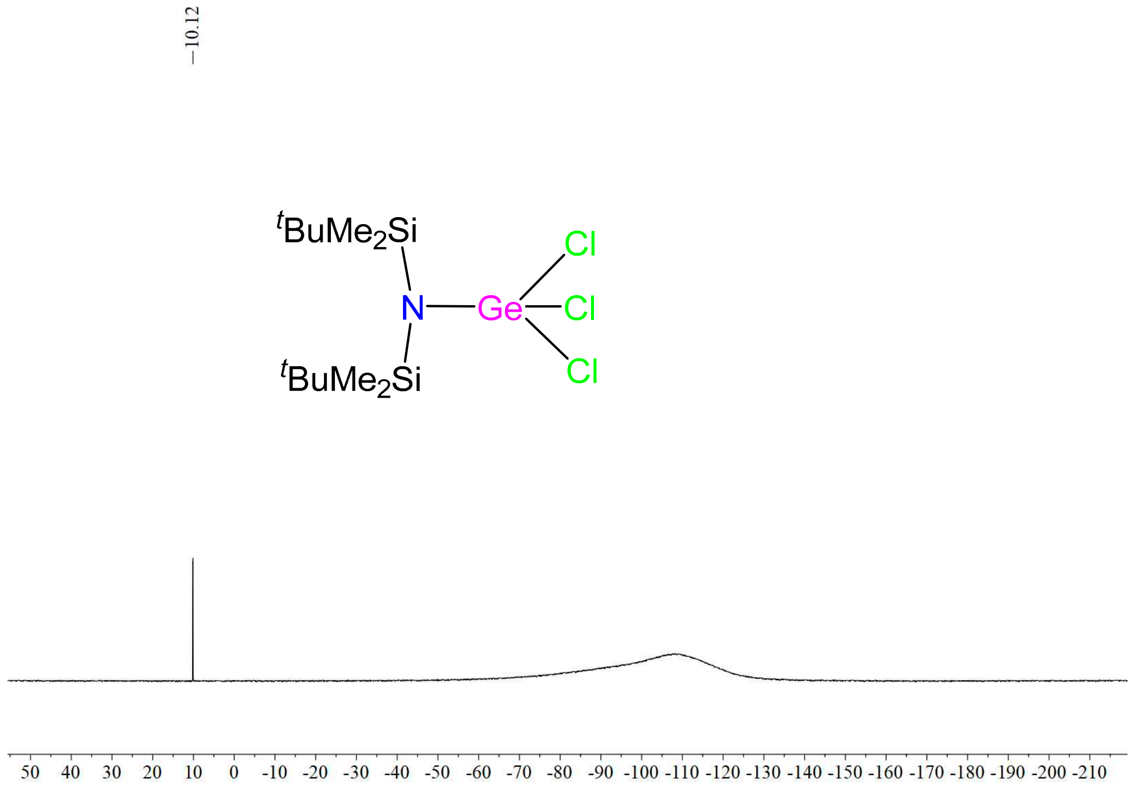

Figure S3. ^{29}Si-NMR (600 MHz) spectrum of **2** in C_6D_6.

Compound 3

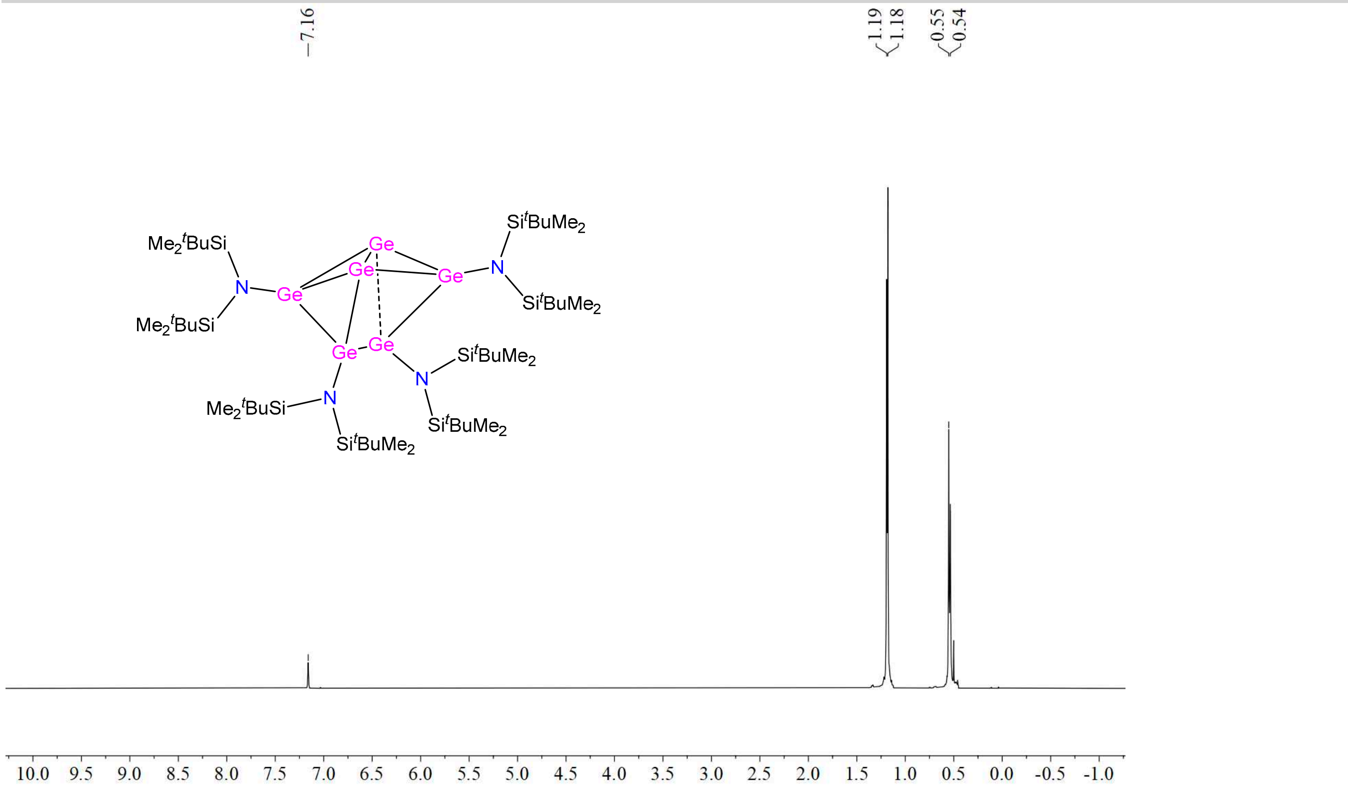

Figure S4. ^{1}H-NMR (400 MHz) spectrum of **3** in C_6D_6.

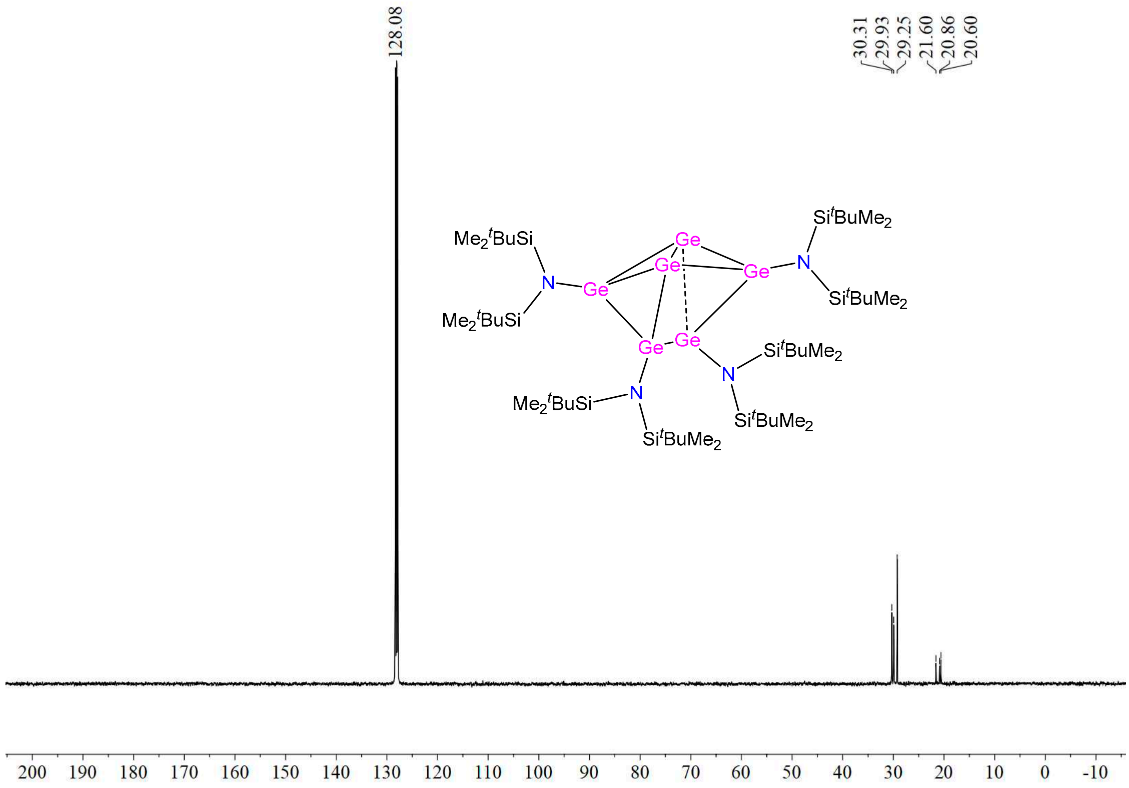

Figure S5. ^{13}C-NMR (100 MHz) spectrum of **3** in C_6D_6.

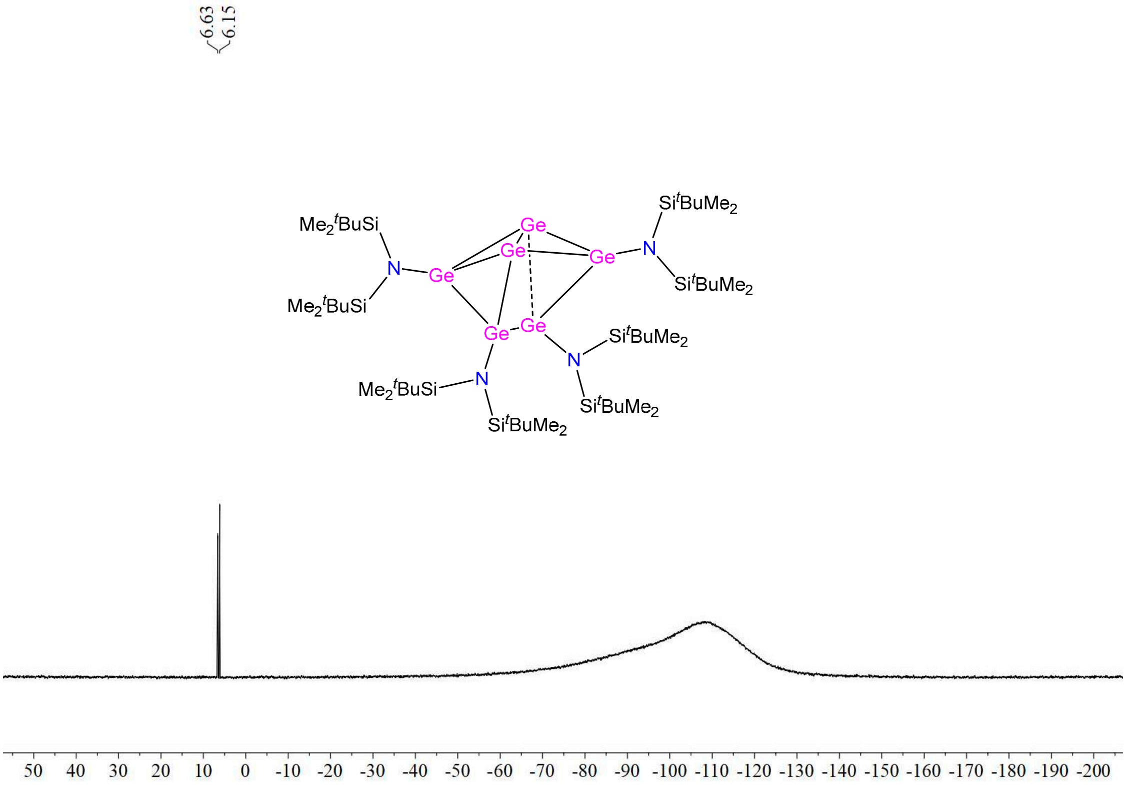

Figure S6. ^{29}Si-NMR (600 MHz) spectrum of **3** in C_6D_6.

Compound 4

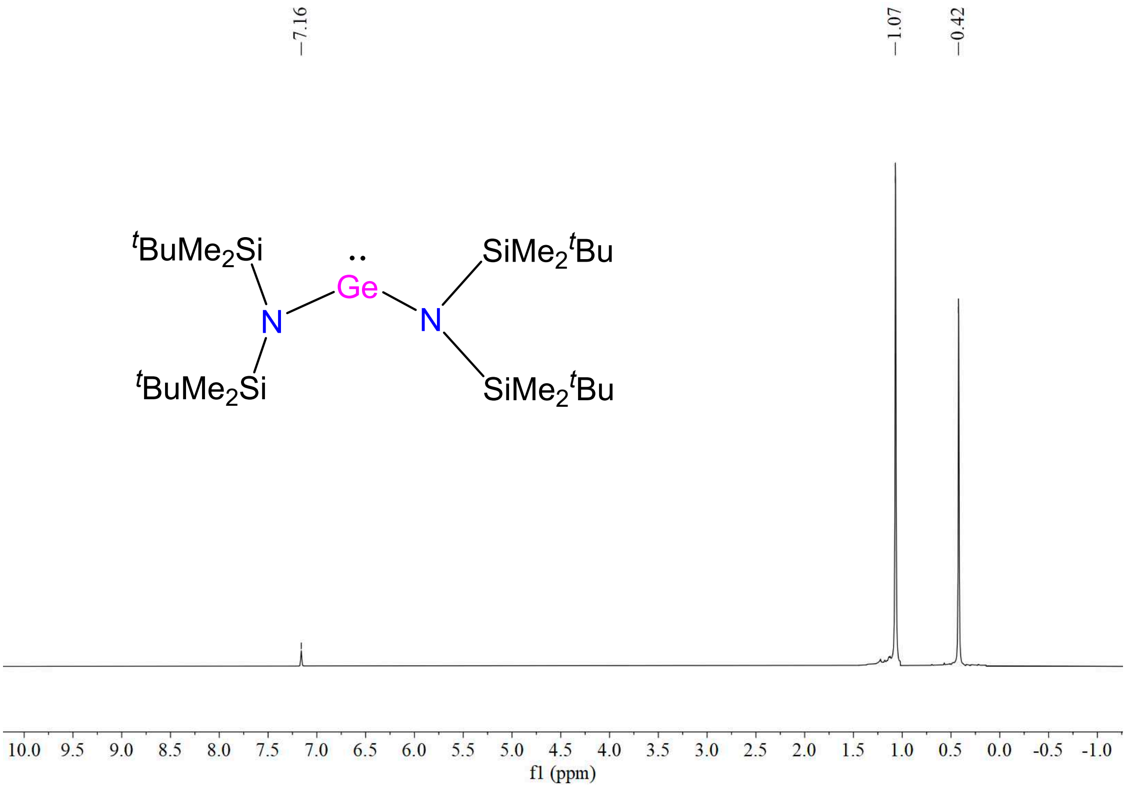

Figure S7. ^{1}H-NMR (400 MHz) spectrum of **4** in C_6D_6.

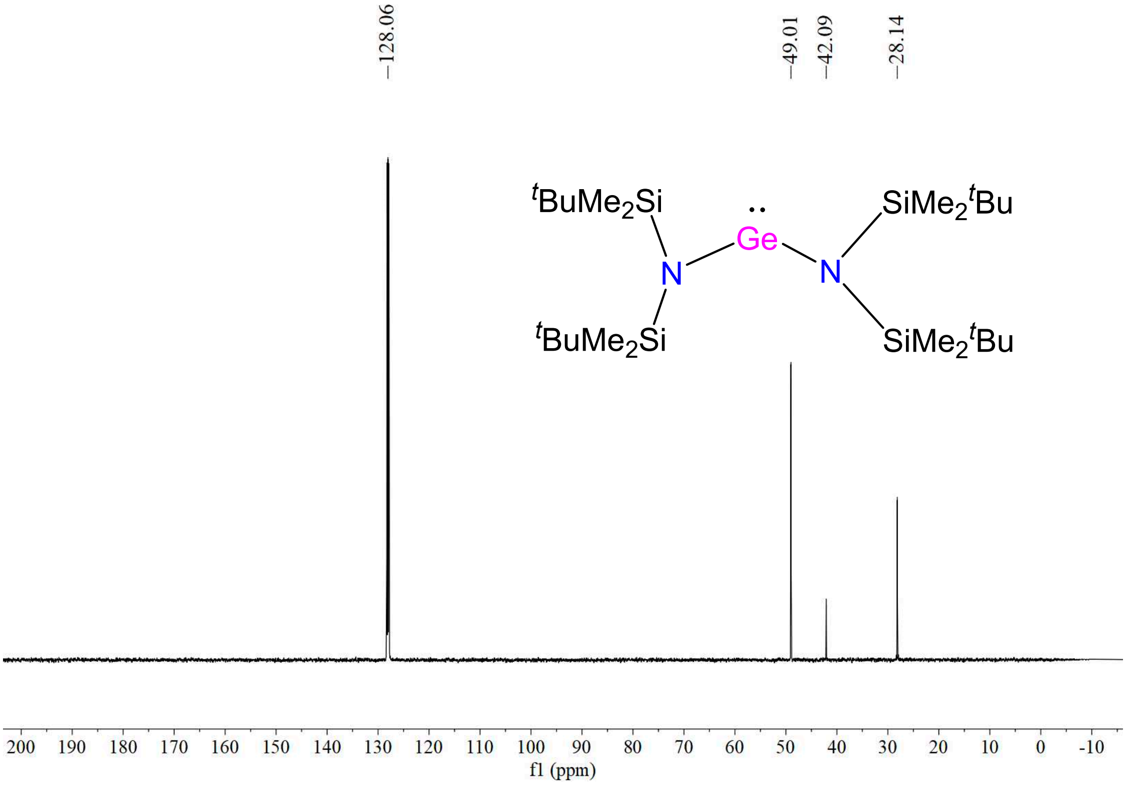

Figure S8. ^{13}C-NMR (100 MHz) spectrum of **4** in C_6D_6.

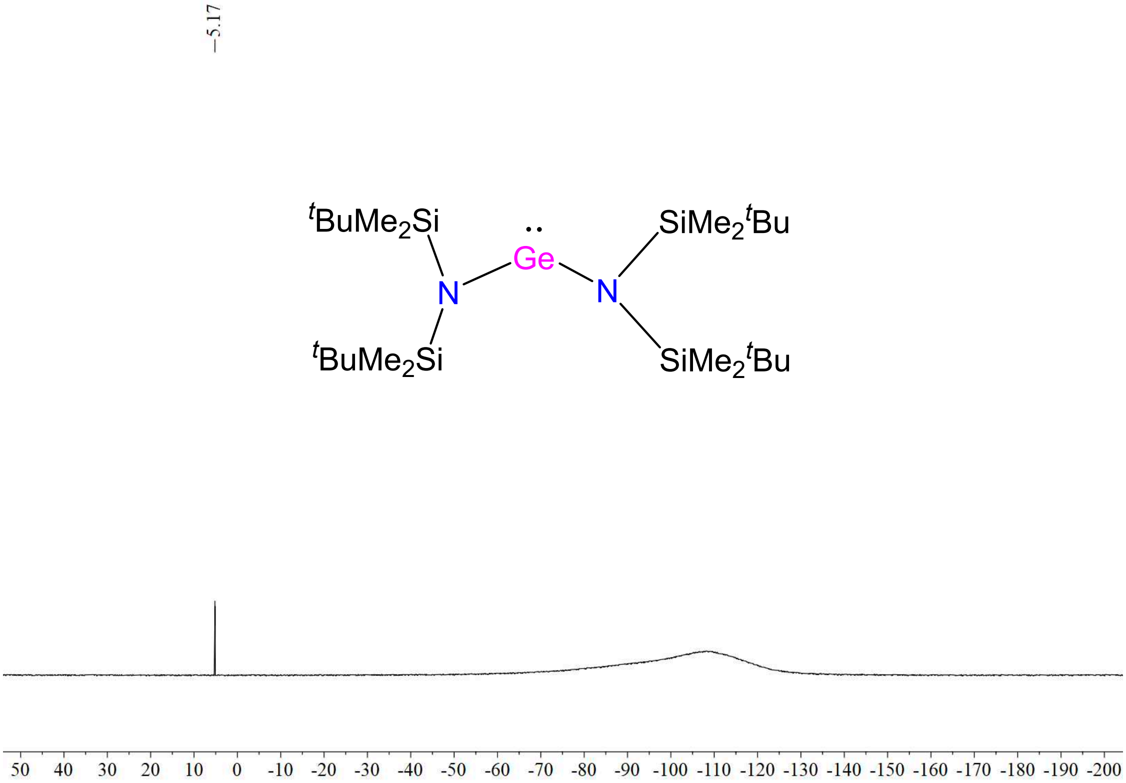

Figure S9. ^{29}Si-NMR (600 MHz) spectrum of **4** in C_6D_6.

Compound 6

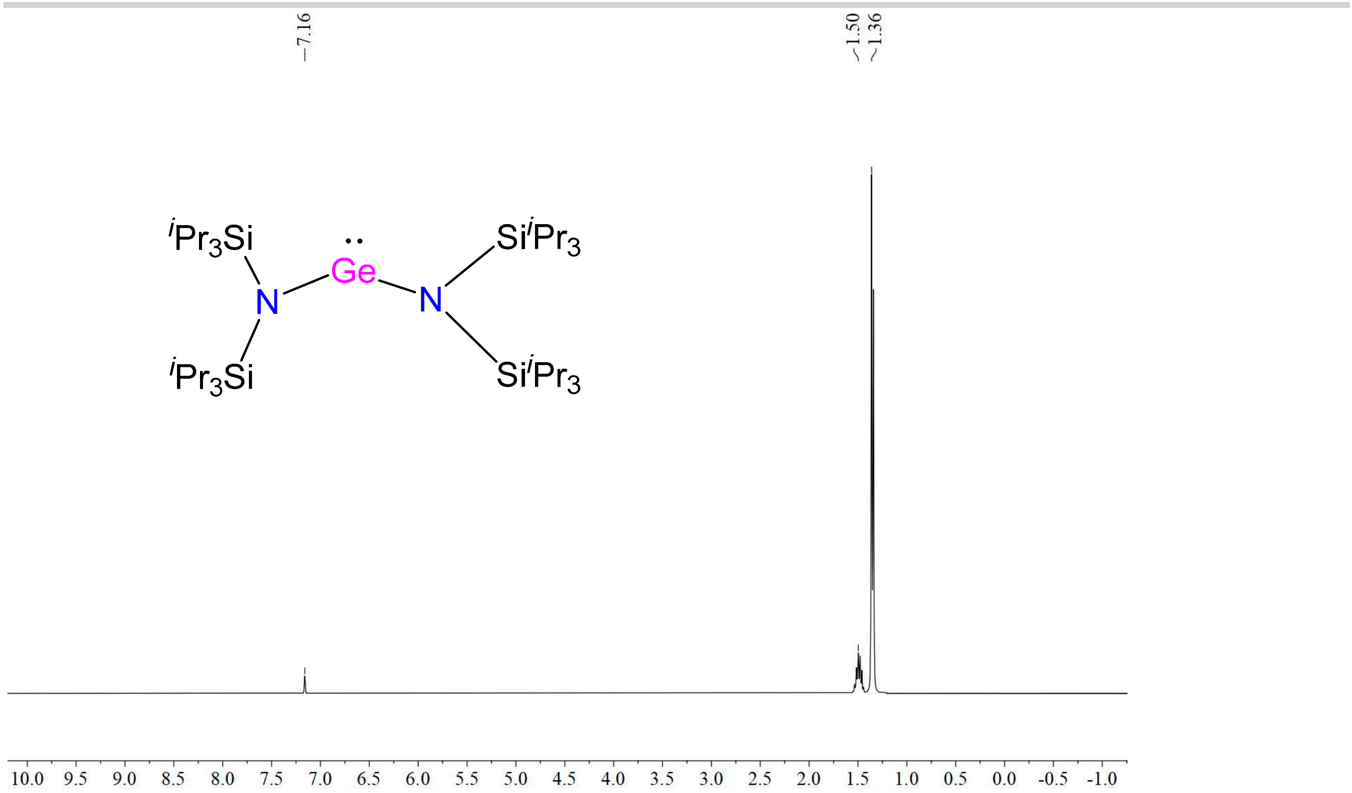

Figure S10. ^{1}H-NMR (400 MHz) spectrum of **6** in C_6D_6.

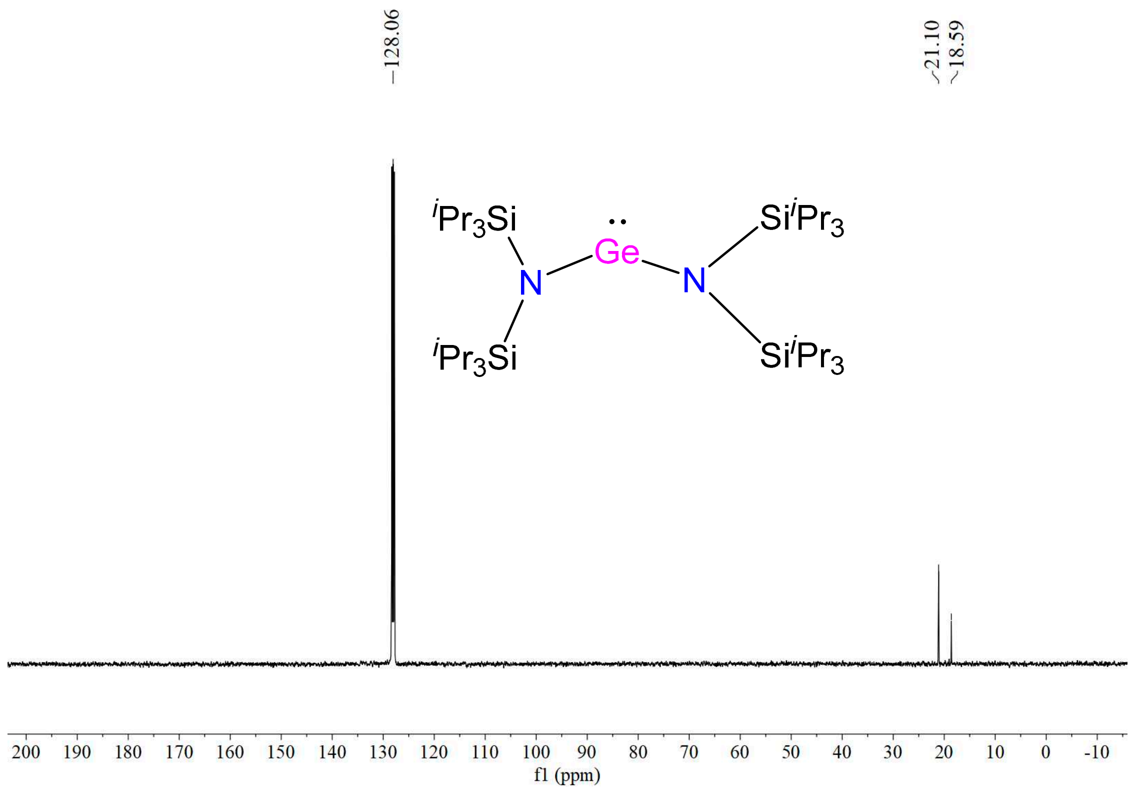

Figure S11. ^{13}C-NMR (100 MHz) spectrum of **6** in C_6D_6.

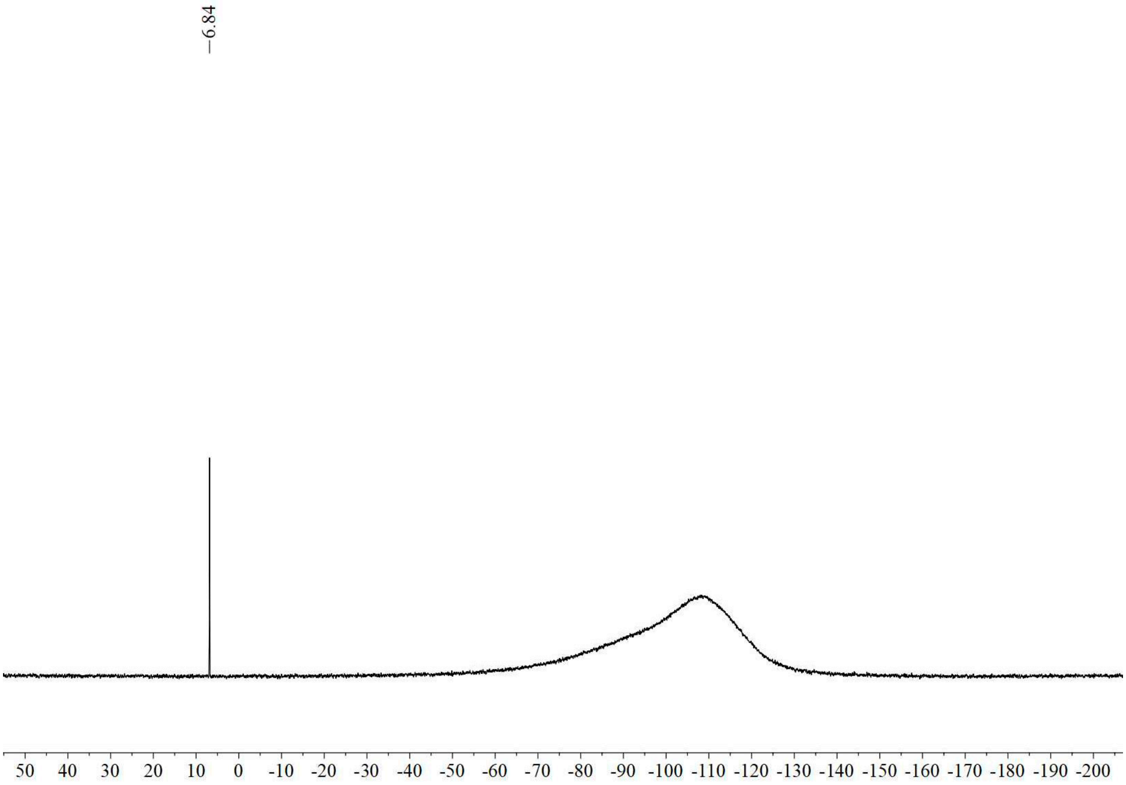

Figure S12. ^{29}Si-NMR (600 MHz) spectrum of **6** in C_6D_6.

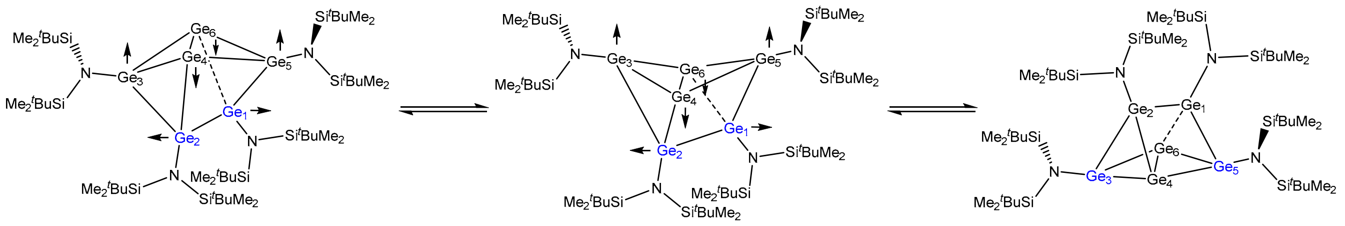

Figure S13. Proposed process for the molecular dynamics of **3**.

D. Computational details

The geometries of **3**, **4** and **6** were optimized by the TPSS/def2-TZVP level of theory [6, 7]. Figure S9-S10 list the selected Intrinsic Bond Orbital of **3**, as well as Natural Bond Orbital (NBO) Charge Distributions of compounds **4** and **6**, the optimized structures are in excellent agreement with single-crystal XRD results of these compounds.

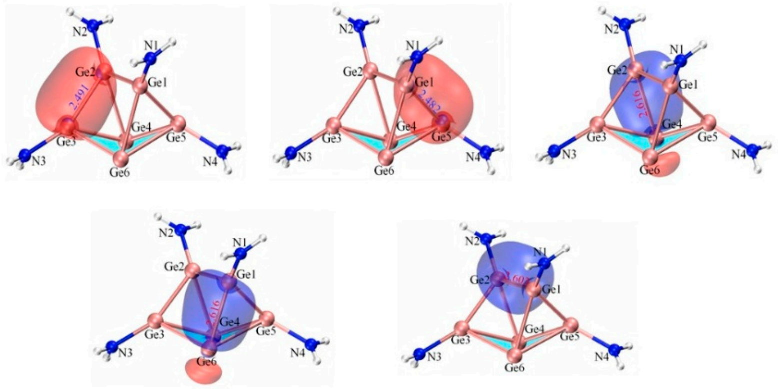

Figure S14. Selected Intrinsic Bond Orbital of **3** at the TPSS/def2-TZVP level.

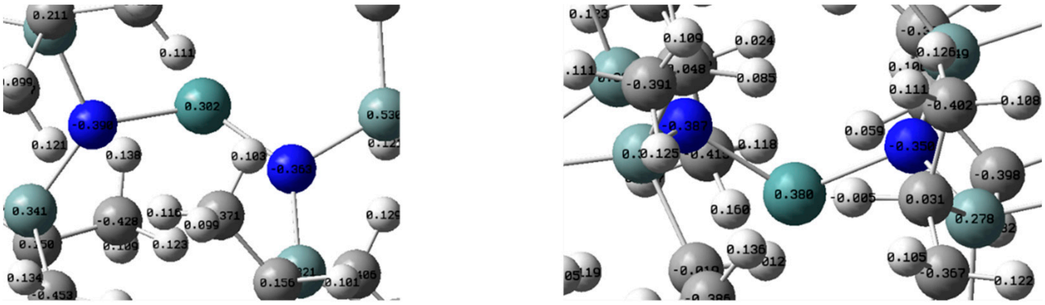

4 6

Figure S15. Natural Bond Orbital (NBO) Charge Distributions of Compounds **4** and **6**.

E. References

1 Lemierre, V.; Chrostowskal, A.; Dargelos, A.; Baylère, P.; Leigh, W. J.; Harrington, C. R. Flash vacuum thermolysis of 3,4-dimethyl-1-germacyclopent-3-enes: UV photoelectron spectroscopic characterization of GeH_2 and $GeMe_2$. *Appl. Organometal. Chem.* **2004**, 18, 676-683.

2 Goodwin, C. A. P.; Joslin, K. C.; Lockyer, S. J.; Formanuik, A.; Morris, G. A.; Ortu, F.; Vitorica-Yrezabal, I. J.; Mills, D. P. Homoleptic Trigonal Planar Lanthanide Complexes Stabilized by Superbulky Silylamide Ligands. *Organometallics* **2015**, 34, 2314-2325.

3 Chilton, N. F.; Goodwin, C. A. P.; Mills, D. P.; Winpenny, R. E. P. The first near-linear bis(amide) f-block complex: a blueprint for a high temperature single molecule magnet. *Chem. Commun.* **2015**, 51, 101-103.

4 Dolomanov, O. V.; Bourhis, L. J.; Gildea, R. J.; Howard, J. A. K.; Puschmann, H. *OLEX2*: a complete structure solution, refinement and analysis program. *J. Appl. Crystallogr.* **2009**, 42, 339−341.

5 Sheldrick, G. M. A short history of SHELX. *Acta Crystallogr. Sect. A* **2008**, 64, 112−122.

6 Perdew, J. P.; Tao, J.; Staroverov, V. N.; Scuseria, G. E. Meta-generalized gradient approximation: Explanation of a realistic nonempirical density functional. *J. Chem. Phys.* **2004**, 120, 6898-6911.

7 Weigenda, F.; Ahlrichsb, R. Balanced basis sets of split valence, triple zeta valence and quadruple zeta valence quality for H to Rn: Design and assessment of accuracy. *Phys. Chem. Chem. Phys.* **2005**, 7, 3297-3305.
